# SCCA1/SERPINB3 suppresses antitumor immunity and blunts therapy-induced T cell responses via STAT-dependent chemokine production

**DOI:** 10.1172/JCI163841

**Published:** 2023-08-01

**Authors:** Liyun Chen, Victoria Shi, Songyan Wang, Lulu Sun, Rebecca Freeman, Jasmine Yang, Matthew J. Inkman, Subhajit Ghosh, Fiona Ruiz, Kay Jayachandran, Yi Huang, Jingqin Luo, Jin Zhang, Pippa Cosper, Clifford J. Luke, Catherine S. Spina, Perry W. Grigsby, Julie K. Schwarz, Stephanie Markovina

**Affiliations:** 1 Department of Radiation Oncology,; 2 Department of Pathology and Immunology,; 3 Department of Surgery, Division of Public Health Sciences, and; 4 Alvin J Siteman Cancer Center, Washington University School of Medicine, St Louis, Missouri, USA.; 5 Department of Human Oncology, University of Wisconsin School of Medicine and Public Health, Madison, Wisconsin, USA.; 6 Department of Pediatrics, Washington University School of Medicine, St Louis, Missouri, USA.; 7 Department of Radiation Oncology, Columbia University Irving Medical Center, New York, New York, USA.; 8 Department of Radiology, Washington University School of Medicine, St Louis, Missouri, USA.

**Keywords:** Oncology, Cervical cancer, Radiation therapy

## Abstract

Patients with cancer who have high serum levels of squamous cell carcinoma antigen 1 (SCCA1, now referred to as SERPINB3) commonly experience treatment resistance and have a poor prognosis. Despite being a clinical biomarker, the modulation of SERPINB3 in tumor immunity is poorly understood. We found positive correlations of *SERPINB3* with *CXCL1*, *CXCL8* (*CXCL8/9*), *S100A8*, and *S100A9* (*S100A8*/*A9*) myeloid cell infiltration through RNA-Seq analysis of human primary cervical tumors. Induction of SERPINB3 resulted in increased CXCL1/8 and S100A8/A9 expression, which promoted monocyte and myeloid-derived suppressor cell (MDSC) migration in vitro. In mouse models, *Serpinb3a* tumors showed increased MDSC and tumor-associated macrophage (TAM) infiltration, contributing to T cell inhibition, and this was further augmented upon radiation. Intratumoral knockdown (KD) of *Serpinb3a* resulted in tumor growth inhibition and reduced CXCL1 and S100A8/A expression and MDSC and M2 macrophage infiltration. These changes led to enhanced cytotoxic T cell function and sensitized tumors to radiotherapy (RT). We further revealed that SERPINB3 promoted STAT-dependent expression of chemokines, whereby inhibition of STAT activation by ruxolitinib or siRNA abrogated CXCL1/8 and S100A8/ A9 expression in SERPINB3 cells. Patients with elevated pretreatment SCCA levels and high phosphorylated STAT3 (p-STAT3) had increased intratumoral CD11b^+^ myeloid cells compared with patients with low SCCA levels and p-STAT3, who had improved overall survival after RT. These findings provide a preclinical rationale for targeting SERPINB3 in tumors to counteract immunosuppression and improve the response to RT.

## Graphical abstract

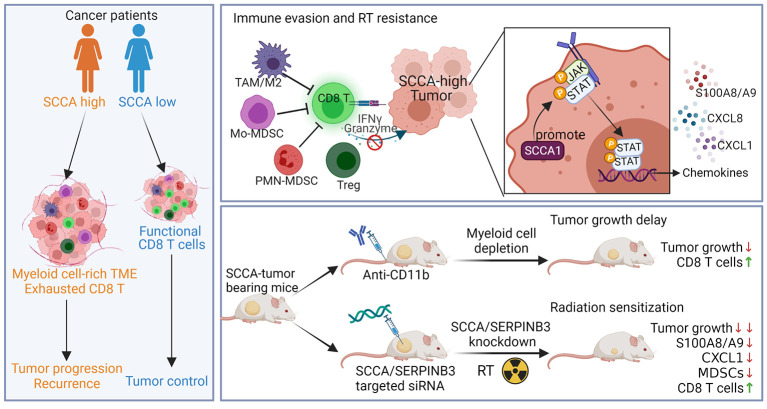

## Introduction

Radiotherapy (RT) is commonly used for the treatment of patients with squamous cell carcinomas, including head and neck, esophageal, lung, and cervical cancers (1). RT can have both immunostimulatory and immunosuppressive effects, which in part determines the prognosis of cancer (1). The activation and infiltration of cytotoxic T cells after radiation is critical to the curative activity of RT. However, tumors with an immunosuppressive tumor microenvironment (TME), dominated by myeloid cells, such as polarized M2 macrophage and myeloid-derived suppressor cells (MDSCs), tend to diminish T cell activity and may be more susceptible to the suppressive immune response induced by RT (2, 3). Chemokines are a subclass of cytokines with chemotactic properties that control the migration of cells and influence the composition of the tumor immune microenvironment (4). Some chemokines, such as CXCL9, CXCL10, CXCL11, CXCL16, promote an immunostimulatory environment, which improves DC activation and T cell trafficking to tumors (4, 5). Conversely, CCL2, CCL5, CXCL1, CXCL8, and CXCL12 can be induced by RT and have the opposite effect of recruiting suppressive immune cells and inhibiting effector T cells, and often correlate with poor treatment outcomes (6–8).

Squamous cell carcinoma antigen 1 (SCCA1), encoded by the *SERPINB3* gene locus and now known as SERPINB3, is a highly conserved cysteine proteinase inhibitor that interacts with lysosomal proteases upon lysosomal leakage and prevents cell death (9). We recently demonstrated that SERPINB3 also protects cervical tumor cells against RT-induced cell death by preventing lysoptosis (10). In many cancers, SERPINB3/SCCA (the ELISA-based clinical assay used to measure circulating SERPINB3 is still referred to as “SCCA”) is highly expressed in tumors or in the circulation of patients with cancer, including cervical, head and neck, lung, breast, and esophageal cancers, and is often associated with poor prognosis and treatment outcomes and disease recurrence (11–15). In addition, elevated SERPINB3 expression is also found in autoimmune disorders and implicated in the induction of inflammatory cytokines (16). However, in both tumors and autoimmune diseases, the mechanistic link between SERPINB3 and immune regulation remains poorly understood. Considering the increasing number of studies reporting the association of SERPINB3 with tumorigenesis (17), metastasis (18), prognosis, and recurrence, additional roles of SERPINB3, independent of proteinase-inhibitory activity, in tumor progression and resistance to therapy are likely.

We have previously demonstrated that patients with persistently high levels of SCCA before treatment and throughout the course of definitive RT had an increased risk of recurrence and death (14). Prospective cohort studies also showed the prognostic value of SCCA for monitoring the response to RT and post-RT clinical outcomes for patients with cervical cancer (19). Given the unfavorable outcomes of patients with high SERPINB3 expression levels, we hypothesized that SERPINB3 promotes immune evasion by modulating suppressive immune responses that alter the TME and impede RT-induced antitumor immunity. Our data showed that SERPINB3 tumors secreted high levels of chemokines that attract myeloid cells. These myeloid cell populations in SERPINB3 tumors possessed potent immunosuppressive activity and inhibited T cell activation, leading to a RT-resistant environment. Targeting CD11b^+^ myeloid cells or SERPINB3 both reduced tumor growth, however, the latter in combination with RT demonstrated more sustained inhibition of tumor growth and remodeling of infiltrating myeloid cells. We further discovered that STAT signaling played an essential role in inducing expression of immune-suppressive chemokines in SERPINB3-expressing cells. Patients with cervical cancer with high SERPINB3/SCCA expression had increased expression of phosphorylated STAT3 (p-STAT3) and CD11b. Here, we identify a regulatory function of SERPINB3 in establishing a protumor microenvironment and the clinical importance of targeting SERPINB3 to improve RT-induced antitumor immunity.

## Results

### SERPINB3 tumors are marked by a myeloid cell–rich and suppressive immune profile.

**Figure 1:**
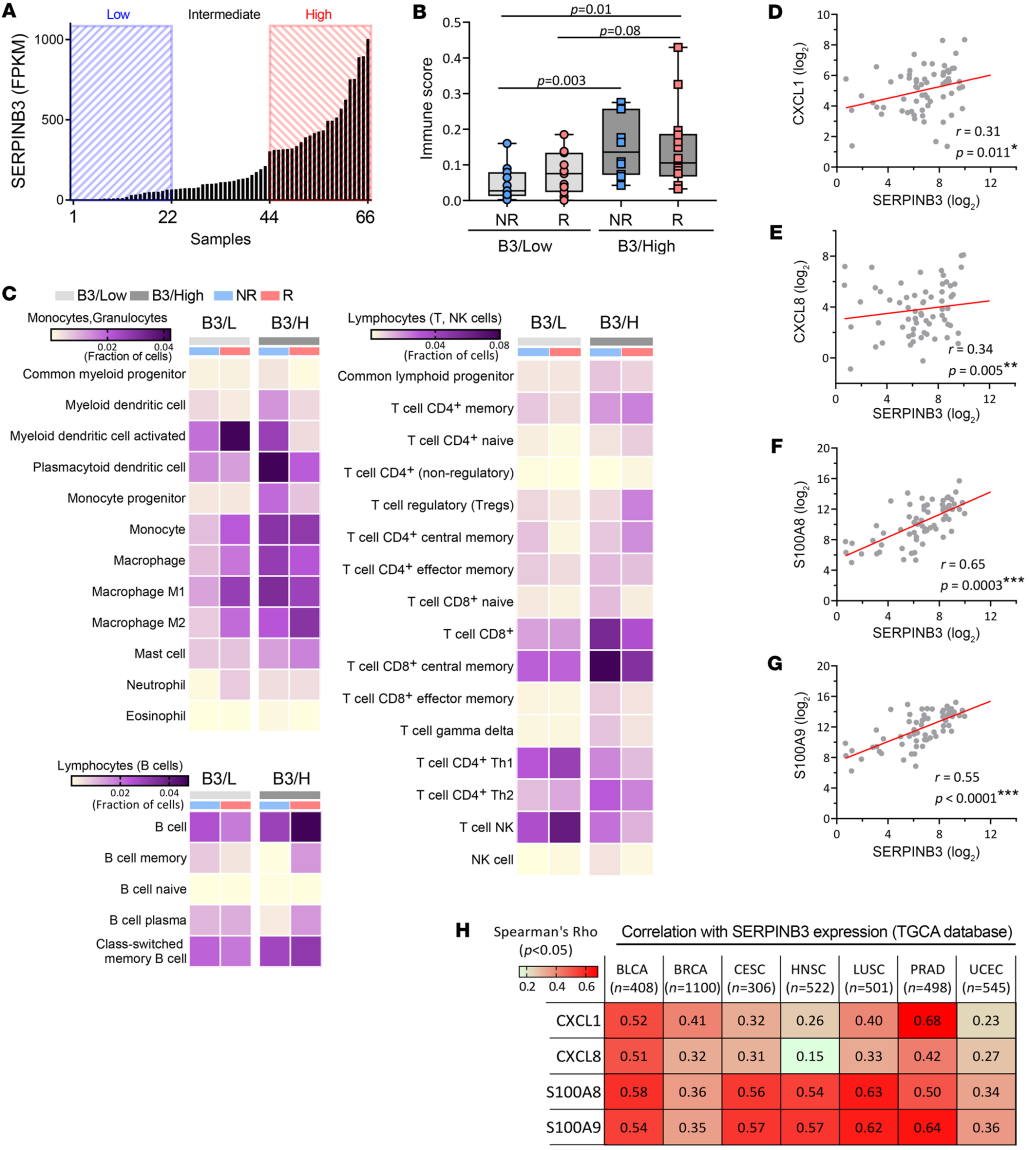
SERPINB3 tumors are marked by a myeloid cell–rich and immune-suppressive profile. (**A**) Normalized SERPINB3 transcripts in cervical tumor biopsies from RNA-Seq were distributed by reads per kilobase of transcript per million mapped reads (RPKM). (**B**) Box plots along with individual data points show xCell immune scores in recurrent (R)/nonrecurrent (NR) B3/L and B3/H tumors. *P < 0.05, by 1-way ANOVA. (**C**) A heatmap of enriched immune cell subpopulations was generated through xCell immune infiltrate prediction. The color intensity is proportional to the average xCell score for each cell population across samples. (**D**–**G**) Spearman’s correlation of SERPINB3 with the expression of (**D**) CXCL1, (**E**) CXCL8, (**F**) S100A8, and (**G**) S100A9 from RNA-Seq of 66 cervical tumor biopsies collected prior to (chemo)-RT. (**H**) SERPINB3 expression correlated with CXCL1, CXCL8, S100A8, and S100A9 expression in multiple cancer types. Analysis was performed using TCGA PanCancer Atlas, and numeric values indicate Spearman’s correlation coefficient. BLCA, bladder urothelial carcinoma; BRCA, breast invasive carcinoma; HNSC, head and neck squamous cell carcinoma; LUSC, lung squamous cell carcinoma; PRAD, prostate adenocarcinoma; UCEC, uterine corpus endometrial carcinoma.

RNA-Seq was performed on 66 cervical tumor biopsies collected prior to chemoradiotherapy (CRT). The patient and tumor characteristics of this cohort have been previously described and are summarized in Supplemental Table 1; supplemental material available online with this article; https://doi.org/10.1172/JCI163841DS1 Patients were divided into 3 groups on the basis of their distribution of *SERPINB3* transcript levels: SERPINB3-low (B3/L, *n* = 22), SERPINB3-intermediate (B3/Int, *n* = 22), and SERPINB3-high (B3/H, *n* = 22) groups (Figure 1A). To investigate the distinct immune signature associated with *SERPINB3* expression in tumors, we focused our analysis on the B3/L versus B3/H patient groups. The immune score (IS) was determined using xCell (20) via gene signature–based, single-sample gene set enrichment analysis, with the overall score representing a ranking of tumors in the data set by lowest (IS of 0) to highest immune infiltrate. B3/H tumors showed overall higher ISs than did B3/L tumors, and this was true for patients whose cancer recurred or did not recur, indicating enrichment of infiltrating immune cells in the TME of B3/H tumors (Figure 1B and Supplemental Figure 1A). Immune cell content showed that B3/H tumors were characterized by increased myeloid cell subsets, including macrophages, monocytes, plasmacytoid DCs, and a small subset of CD8^+^ T lymphocytes. In contrast, there were fewer T helper type 1 (Th1), Th2, and NK T cells in B3/H compared with B3/L tumors (Figure 1C and Supplemental Figure 1B).

We then investigated the differential expression of 2 major human chemokine subfamilies, CC and CXC chemokines, among the 3 groups (Supplemental Figure 1C). Expression levels of 2 chemokines, *CXCL1* and *CXCL8*, which are associated with the recruitment of myeloid cells, correlated with *SERPINB3* expression (Figure 1, D and E). In contrast, expression of the T cell– and NK cell–recruiting chemokines *CXCL9*, *CXCL10*, and *CXCL16* was not associated with *SERPINB3* expression (Supplemental Figure 1C). Further analysis of chemokines that attract myeloid cells demonstrated a positive correlation between *SERPINB3* and *S100A8/S100A9* expression (Figure 1, F and G). These correlations were validated in The Cancer Genome Atlas–Cervical Squamous Cell Carcinoma and Endocervical Adenocarcinoma (TCGA-CESC) (*n* = 306) data set (Supplemental Figure 1D). Notably, analysis of TCGA Pan-Cancer Atlas showed a consistent positive correlation between *SERPINB3* and *CXCL1*, *CXCL8*, *S100A8*, and *S100A9* across multiple tumor types including bladder, breast, head and neck, lung, prostate, and uterine cancers (Figure 1H). These same tumor types have high levels of SERPINB3 expression (Supplemental Figure 1E).

Of note, the HPV subtype was varied in the 66-patient RNA-Seq cohort (Supplemental Table 1), and there was no obvious correlation between HPV^+^ or HPV^–^ tumors and HPV subtype (Supplemental Figure 1F). This is consistent with our previous finding that both HPV^+^ and HPV^–^ tumors and tumor cell lines express SERPINB3 (14).

### SERPINB3 results in the upregulation of CXCL1/8 and S100A8/A9 chemoattractants, promoting myeloid cell migration from patient-derived peripheral blood.

**Figure 2:**
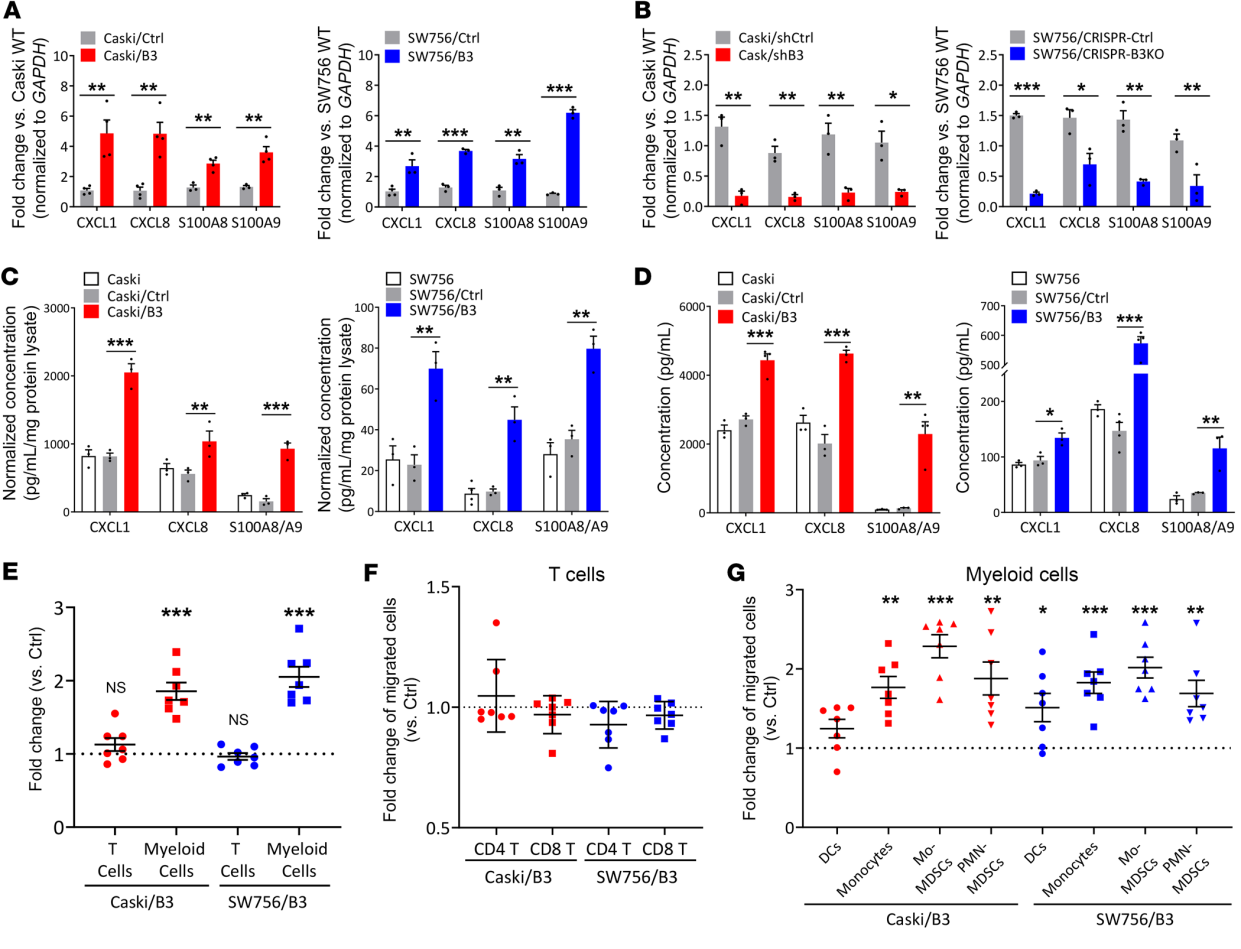
SERPINB3 results in upregulation of CXCL1/8 and S100A8/A9 chemoattractants, promoting myeloid cell migration from patient-derived peripheral blood. (**A**) Cells were transduced with pUltra vector (Caski/Ctrl, SW756/Ctrl) or pUltra-SERPINB3 (Caski/B3, SW756/B3), and CXCL1/8 and S100A8/A9 expression was examined by qPCR. (**B**) Caski cells were transfected with scrambled negative control shRNA (Caski/shCtrl) or shRNAs specifically against SERPINB3 (Caski/shB3); SW756 cells were transduced with a CRISPR control vector (SW756/CRISPR-Ctrl) or CRISPR/Cas9 for SERPINB3 KD (SW756/CRISPR-B3KO). CXCL1/8 and S100A8/A9 expression was examined by qPCR. Gene expression was normalized to *GAPDH*, and fold changes were calculated by comparing with expression levels in parental cells (Caski WT or SW756 WT). (**C**) Intracellular chemokine protein expression was measured by ELISA, and expression levels were normalized to the total protein concentration. (**D**) Supernatant was collected from adherent cells in the monolayer, and chemokine secretion was measured by ELISA. Data are presented as the mean ± SEM of 3 independent experiments. *P < 0.05, **P < 0.01, and ***P < 0.001, by Mann-Whitney *U* test (**A** and **B**) and 1-way ANOVA with Tukey’s post hoc test (**C** and **D**). (**E**–**G**) PBMC migration toward supernatant collected from cancer cells was examined by Transwell assays, and the migrated PBMC populations were analyzed by flow cytometry (Supplemental Figure 3A). Fold changes were calculated as the percentage of migrated (**E**) T cells and myeloid cells, (**F**) T cell subsets, and (**G**) myeloid cell subsets in Caski/B3 or SW756/B3 supernatant relative to Caski/Ctrl or SW756/Ctrl supernatant. Data are shown as the mean ± SEM. *P < 0.05, **P < 0.01, and ***P < 0.001, by 2-tailed, 1-sample *t* test against 1. Each dot represents the mean of duplicate values for a single donor sample (*n* = 7).

To study the mechanistic link between SERPINB3 and chemokine expression, we genetically altered SERPINB3 levels in Caski and SW756 human cervical cancer cells (Supplemental Figure 2A) and examined the effect on chemokine production. Caski and SW756 cells with stable expression of SERPINB3 (Caski/B3, SW756/B3) showed increased CXCL1/8 and S100A8/A9 gene expression (Figure 2A), while downregulation of SERPINB3 expression using shRNA-mediated knockdown (KD) (Caski/shB3) or CRISPR/Cas9-mediated deletion (SW756/CRISPR-B3KO) significantly reduced CXCL1/8 and S100A8/A9 expression (Figure 2B), when compared with their control counterparts. In addition to gene expression, we detected significantly higher CXCL1/8 and S100A8/A9 protein expression and secretion in Caski/B3 versus Caski control (Caski/Ctrl) cells as well as SW756/B3 versus SW756/Ctrl cells (Figure 2, C and D). Because Caski and SW756 cells are positive for HPV16 and HPV18, respectively, whether SERPINB3-induced chemokine expression is associated with HPV infection was examined using the HPV^–^ cervical cancer cells C33A. Similar to the observation in HPV^+^ cells, C33A with SERPINB3 upregulation (C33A/B3) showed increased CXCL1/8 and S100A8/A9 expression (Supplemental Figure 2B). We next examined the chemotactic response of human PBMCs, obtained from 7 patients with biopsy-proven cervical cancer prior to delivery of any treatment, to the chemokines secreted by tumor cells with high SERPINB3 expression. Supernatant collected from Caski/B3 and SW756/B3 cells promoted the migration of CD11b^+^ myeloid cells, with an average 1.9-fold increase in Caski/B3 versus Caski/Ctrl supernatant and 2.1-fold increase in SW756/B3 versus SW756/Ctrl supernatant, whereas the migration of CD4^+^ and CD8^+^ T cells showed no statistical difference (Figure 2, E and F). Further analysis of migrated CD11b^+^ cells showed that cell populations migrating in response to both Caski/B3 and SW756/B3 supernatants were enriched in monocytes, and monocytic and polymorphonuclear MDSCs (M-/PMN-MDSCs), with an approximately 1.5- to 2-fold increase compared with Ctrl supernatant cells (Figure 2G and Supplemental Figure 3, B and C).

### SERPINB3 tumors show accumulated myeloid cells and increased tumor growth.

Since SERPINB3 upregulated the expression of myeloid chemoattractants in vitro*,* we hypothesized that tumors expressing SERPINB3 attract myeloid cell infiltration and mediate the in vivo TME. Human Caski/Ctrl or Caski/B3 cells were injected subcutaneously into the flank of athymic nude mice, and tumor-infiltrating immune cells were analyzed (Supplemental Figure 4A). Tumor growth showed no difference between Caski/Ctrl and Caski/B3 tumors over the course of the experiment (Supplemental Figure 4B); however, Caski/B3 tumors had a significant increase in infiltrating CD11b^+^ myeloid cells compared with Caski/Ctrl tumors at days 22 and 40 after injection (Supplemental Figure 4C). M-MDSCs, tumor-associated macrophages (TAMs), and M2 macrophages were significantly increased in Caski/B3 tumors at both days 22 and 40, while no difference was seen in DCs, PMN-MDSCs, or B cells (Supplemental Figure 4D).

Given that lymphocyte-mediated immune activity plays a role in tumor response to RT and that RT is known to reshape the TME, the SERPINB3-mediated TME and its response to radiation was characterized in an immunocompetent murine model. However, to our knowledge, there are no murine cervical tumor cell lines, and the commonly used alternative, TC1 cells with HPV E6/E7 gene expression, were derived from normal lung epithelial cells with relatively low chemokine expression (Supplemental Figure 5A). Therefore, constructs driving murine *Serpinb3a*, homologous to human *SERPINB3* (21), were expressed in LL2 murine lung carcinoma cells (LL2/B3a), and an empty vector was used as a control (LL2/Ctrl) (Supplemental Figure 5B). Of note, SERPINB3 is also expressed in lung cancer (Supplemental Figure 1C) and is negatively associated with prognosis, providing credence to this model (11). The expression of murine CXCL1 and CXCL3, functionally corresponding to human CXCL1/8, and murine S100A8 and S100A9 (S100A8/A9), homologous to human S100A8/A9, was induced by *Serpinb3a*, whereas the chemokines *Cxcl9* and *Cxcl10*, which are associated with T cell migration, were not affected by *Serpinb3a* expression (Supplemental Figure 5C).

**Figure 3:**
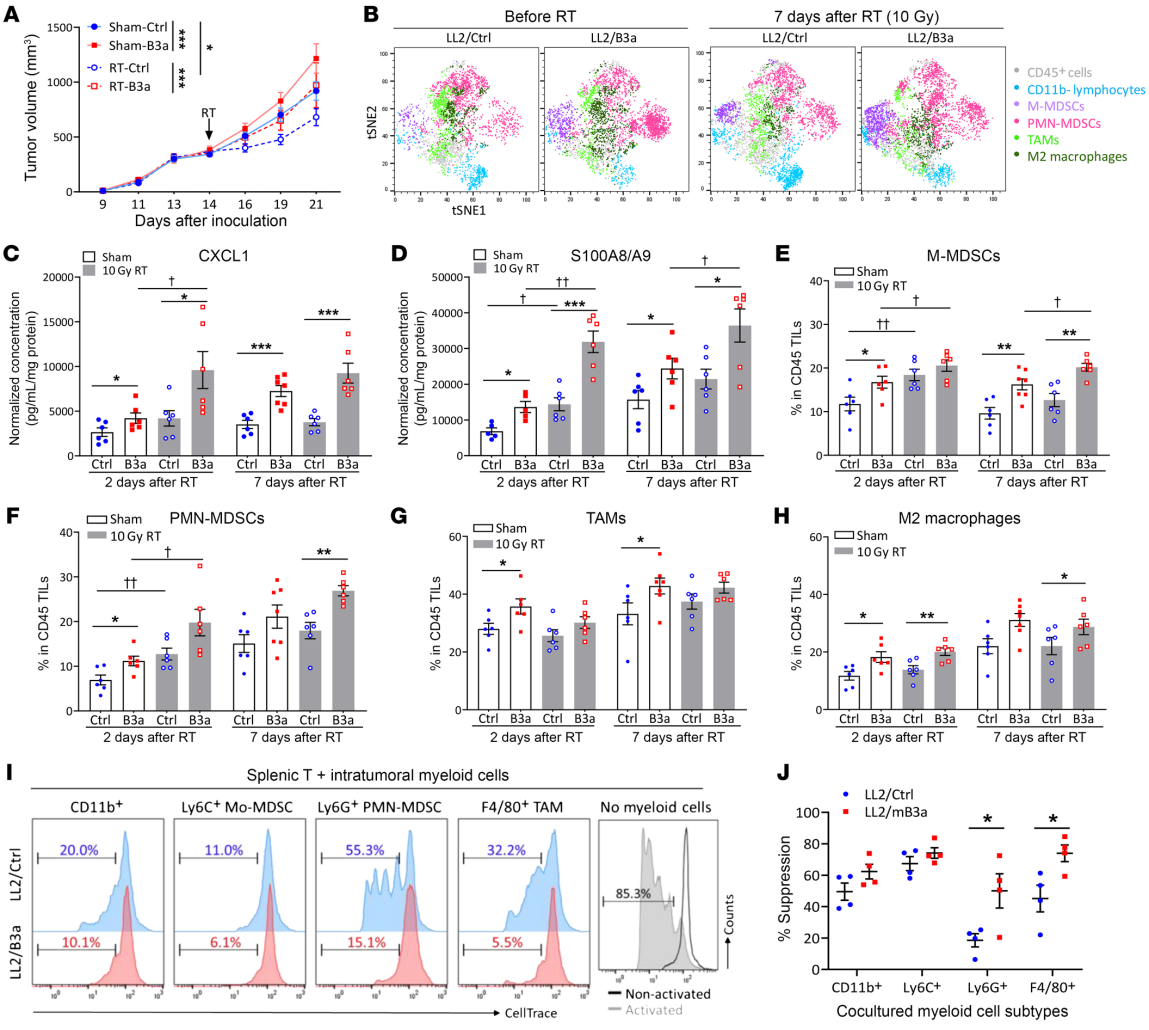
SERPINB3 tumors are enriched for suppressive myeloid cells, and this suppression is further augmented by RT. (**A**) Tumor growth of C57/BL6 mice with LL2/Ctrl tumors (blue lines) and LL2/B3a tumors (red lines) randomized to receive sham treatment (solid lines) or 10 Gy RT on day 14 (dotted lines). *P < 0.05 and ***P < 0.001, by 2-way ANOVA. (**B**) viSNE plots show flow cytometric analysis of total viable CD45^+^ immune cells from tumors with separate clustering by predefined cell-surface markers, including M-MDSCs (CD11b^+^Ly6G^–^Ly6C^hi^), PMN-MDSCs (CD11b^+^Ly6G^+^), TAMs (CD11b^+^Ly6G^–^F4/80^+^), M2 macrophages (CD11b^+^Ly6G^–^F4/80^+^CD163^+^), and lymphocytes (CD45^+^CD11b^–^). (**C** and **D**) The chemokines CXCL1 and S100A8/A9 in tumor homogenates were examined by ELISA. Data were normalized to the protein concentration for each tumor homogenate. (**E**–**H**) Cumulative data from FACS analysis show alteration of immune cell infiltration by SERPINB3 expression and radiation in LL2 tumors. The graphs represent the frequencies of (**E**) CD11b^+^Ly6G^–^Ly6C^hi^ M-MDSCs, (**F**) CD11b^+^Ly6G^+^ PMN-MDSCs, (**G**) CD11b^+^Ly6G-F4/80^+^ TAMs, (**H**) CD11b^+^Ly6G-F4/80^+^CD163^+^ M2 macrophages in total TILs. Data in **C**–**H** are shown as the mean ± SEM, and each dot represents a biologically independent animal; asterisks indicate comparisons between LL2/Ctrl and LL2/B3a; cross symbols indicate comparisons between sham-treated and RT. *,^†^*P* < 0.05, **,^††^*P* < 0.01, and ***P < 0.001, by 1-way ANOVA with Tukey’s post hoc test. (**I** and **J**) Myeloid cell subtypes were isolated from tumors and cocultured with CellTrace-labeled splenic T cells at a ratio of 1:1 for 4 days. Anti-CD3/anti-CD28 antibodies were added to stimulate T cell proliferation. Histograms show the percentage of divided cells. The percentages of suppression were calculated by comparing with the dilution of CellTrace in splenic T cells without myeloid cell coculturing. Data in **I** and **J** are shown as the mean ± SEM. *P < 0.05, by Mann-Whitney *U* test.

LL2/Ctrl and LL2/B3a cells were injected subcutaneously into C57/BL6 mice, which were then randomized to receive a single dose of 10 Gy or sham RT (14 days after injection). Tumor growth curves showed that RT-treated LL2/Ctrl tumors had the smallest volumes, and the RT-treated L2/B3a tumor growth curve overlapped with that of sham-treated LL2/Ctrl tumors, whereas sham-treated LL2/B3a tumors showed the fastest growth (Figure 3A). This is consistent with our prior study showing that human cervical tumor cell lines expressing SERPINB3 are more radioresistant than control tumors in an athymic nude murine model (10). Tumor weights showed no statistical differences between any of the groups 2 days after RT, whereas a more substantial increase in sham- and RT-treated LL2/B3a tumor growth corresponded to increased tumor weights 7 days after RT compared with the LL2/Ctrl counterpart (Supplemental Figure 6A). The visualization of *t*-distributed stochastic neighbor embedding (viSNE) plots show unsupervised clustering of CD45^+^ immune cell subsets based on predefined markers in LL2/Ctrl and LL2/B3a tumors (Supplemental Figure 6B). The viSNE analysis revealed that LL2/B3a tumors had an overall higher number of M-/PMN-MDSCs than did LL2/Ctrl tumors at both pre-RT and post-RT time points. Both irradiated LL2/Ctrl and LL2/B3a tumors had increased numbers of total CD11b^+^ myeloid cells, but different subsets were represented (Figure 3B). Thus, we sought to further examine the dynamic change in immune cell subsets in LL2/Ctrl and LL2/B3a tumors at different time points.

### SERPINB3 tumors are enriched for suppressive myeloid cells, and this enrichment is further augmented by radiation.

Similar to our in vitro findings, LL2/B3a tumors had higher levels of intratumoral CXCL1 and S100A8/A9 expression over time compared with sham-treated LL2/Ctrl tumors (Figure 3, C and D). Radiation promoted further CXCL1 production in RT-treated LL2/B3a tumors but not in RT-LL2/Ctrl tumors (Figure 3C). Although radiation induced S100A8/A9 expression in both RT-LL2/Ctrl and RT-treated LL2/B3a tumors at day 2 after RT, the magnitude of chemokine induction was greater in RT-treated LL2/B3a tumors than in RT-treated LL2/Ctrl tumors, with an average 2.3-fold and 1.8-fold increase, respectively (Figure 3D). Higher and more persistent expression of immunosuppressive chemokines in the tumor milieu of irradiated LL2/B3a tumors led us to hypothesize that the increased myeloid compartment summarized by viSNE plots differed specifically in immunosuppressive myeloid cell subtypes. Indeed, sham-treated LL2/B3a tumors showed consistently higher infiltration of M-MDSCs and PMN-MDSCs compared with LL2/Ctrl tumors, while radiation induced an early increase of infiltrating M-/PMN-MDSCs at day 2 after RT in both groups. However, MDSCs in irradiated tumors remained elevated compared with sham-treated tumors on day 7 after RT only in RT-LL2/B3a tumors (Figure 3, E and F). The number of infiltrating TAMs and M2 macrophages was higher in sham LL2/B3a tumors than in LL2/Ctrl tumors, with a gradual increase in both groups as the tumors grew, but no statistical change with irradiation in either genetic background (Figure 3, G and H).

To assess the immunosuppressive activity of myeloid cells from LL2/Ctrl and LL2/B3a tumors, we isolated intratumoral CD11b^+^ myeloid cells, Ly6C^+^ M-MDSCs, Ly6G^+^ PMN-MDSCs, and F4/80^+^ TAMs and cocultured them with splenic T cells derived from nontumor-bearing mice. Intratumoral Ly6C^+^ M-MDSCs from both LL2/Ctrl and LL2/B3a tumors demonstrated strong inhibition toward T cell proliferation. Notably, Ly6G^+^ PMN-MDSCs and F4/80^+^ TAMs derived from LL2/B3a tumors had more significant inhibitory effects than did those from LL2/Ctrl tumors (Figure 3, I and J).

### Cytotoxic T cells from SERPINB3 tumors display impaired proliferation and exhausted phenotypes.

**Figure 4:**
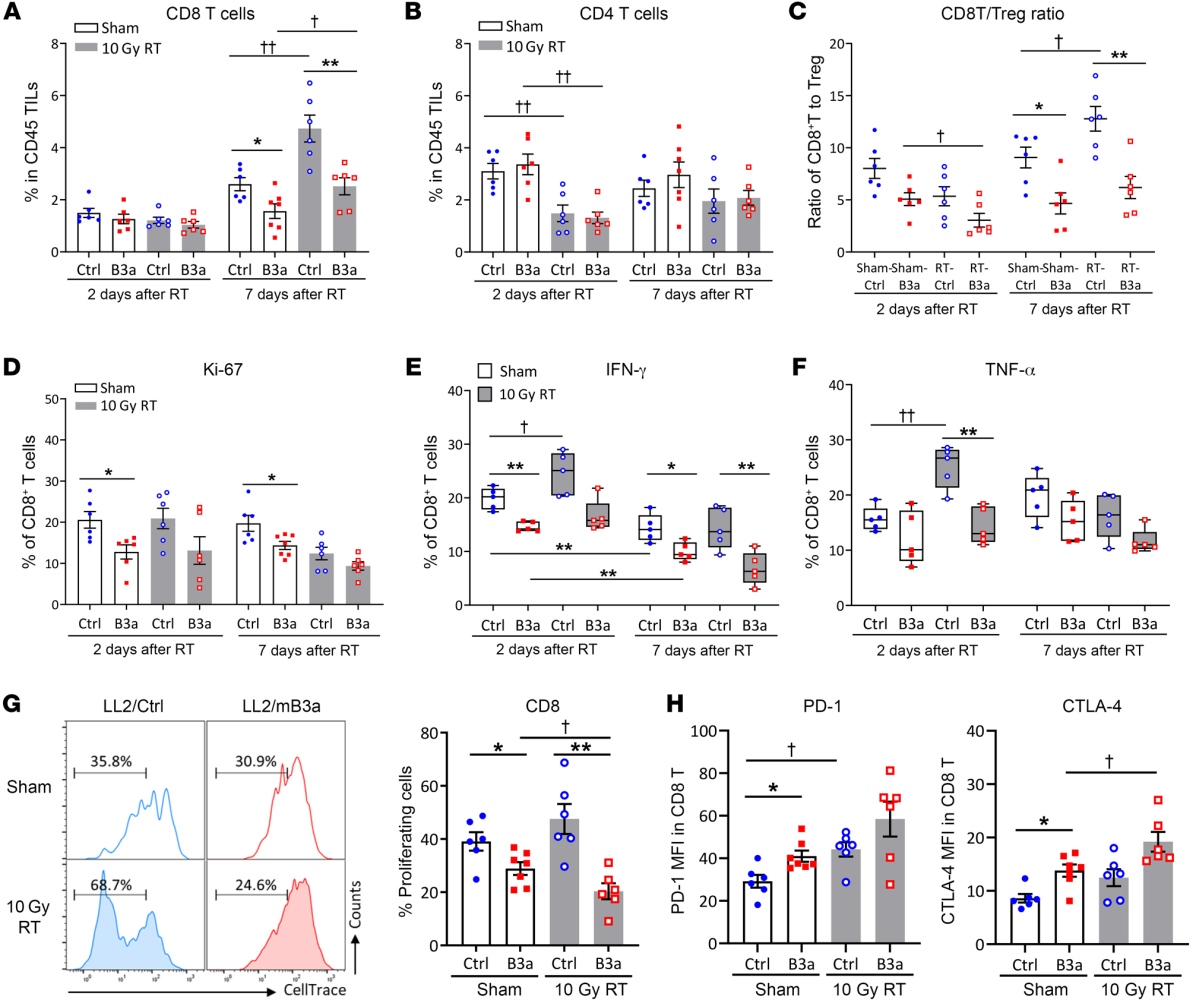
Cytotoxic T cells from SERPINB3 tumors display impaired proliferation and exhausted phenotypes. Cumulative data from FACS analysis of (**A**) CD3^+^CD8^+^ T cells and (**B**) CD3^+^CD4^+^ T cells in tumors. (**C**) The ratio of CD8^+^ T cells/Tregs represented the infiltrating percentage of CD8^+^ T cells relative to CD4^+^CD25^+^FoxP3^+^ Tregs. (**D**) Frequencies of Ki-67^+^CD8^+^ T cells in the total infiltrating CD8^+^ T cell population were analyzed by flow cytometry. (**E** and **F**) Intratumoral T cells were stimulated with phorbol 12-myristate 13-acetate (PMA)/ionomycin for 5 hours, and the expression of IFN-γ and TNF-α was assessed by intracellular staining via flow cytometry. The protein transport inhibitor brefeldin A was used to block the protein transport processes and cytokine release. Positive expression was normalized to cells without PMA/ionomycin stimulation (basal levels). Box plot whiskers span the minimum and maximum values, and lines represent the median. (**G**) CellTrace-labeled intratumoral T cells were stimulated with anti-CD3/anti-CD28 antibody for 4 days, and cell proliferation was determined by the dilution of CellTrace. (**H**) PD-1 and CTLA-4 expression was examined by flow cytometry and is shown as MFI. Data are shown as the mean ± SEM, and each dot represents a biologically independent sample. Asterisks indicate comparisons between LL2/Ctrl and LL2/B3a; cross symbols indicate comparisons between sham- and RT-treated animals. *,^†^*P* < 0.05, **,^††^*P* < 0.01, and ***P < 0.001, by 1-way ANOVA with Tukey’s post hoc test.

With evidence of an immunosuppressive TME, T cell recruitment and function are likely to be compromised in LL2/B3a tumors. CD8^+^ tumor-infiltrating lymphocytes (TILs) numbers were lower in sham- and RT-treated LL2/B3a tumors versus LL2/Ctrl tumors on day 7 after RT. In RT-LL2/Ctrl tumors, CD8^+^ TILs doubled compared with those detected in sham-treated tumors, and while statistically increased, the magnitude of the increase was lower in RT-LL2/B3a tumors (Figure 4A). We observed no difference in CD4^+^ TILs between LL2/Ctrl and LL2/B3a tumors, and a significant decrease on day 2 after RT in both groups was associated with a radiation effect (Figure 4B), consistent with radiosensitivity of in-field lymphocytes (22). The ratio of CD8^+^ T cells to Tregs (CD4^+^CD25^+^FoxP3^+^) was significantly decreased in RT-LL2/B3a compared with sham-LL2/B3a tumors, indicating an increase in Tregs in LL2/B3a tumors shortly after radiation. In contrast, increased CD8^+^ TILs in RT-LL2/Ctrl tumors on day 7 after RT correlated with higher CD8^+^ T/Treg ratios compared with sham-LL2/Ctrl tumors (Figure 4C). Moreover, the proliferation marker Ki-67 showed lower expression in CD8^+^ TILs from LL2/B3a tumors compared with LL2/Ctrl tumors, suggesting that, despite an increased infiltration of CD8^+^ TILs, tumor-directed radiation did not promote the proliferation of CD8^+^ T cells (Figure 4D).

We further evaluated cytotoxic CD8^+^ T cells according to the production of IFN-γ and TNF-α following ex vivo stimulation with PMA and ionomycin. An average of 20% of sham-treated CD8^+^ TILs from LL2/Ctrl tumors and 15% from LL2/B3a tumors showed IFN-γ production, whereas the frequencies of IFN-γ–producing CD8^+^ TILs were reduced with tumor growth in both groups (Figure 4E). CD8^+^ TILs taken from RT-LL2/Ctrl tumors on day 2 after RT showed significant enhancement of both IFN-γ and TNF-α production following stimulation, whereas radiation-boosted IFN-γ and TNF-α production was not observed in CD8^+^ TILs from RT-LL2/B3a tumors (Figure 4, E and F). We examined T cell receptor–mediated (TCR-mediated) activation according to the proliferation of CellTrace-labeled CD8^+^ TILs stimulated with anti-CD3/anti-CD28 antibody. CD8^+^ TILs from sham-treated and RT- LL2/Ctrl tumors demonstrated a stronger proliferative capacity than did those derived from LL2/B3a tumors. Radiation did not have significant effects on TCR-mediated proliferation of CD8^+^ TILs from LL2/Ctrl tumors, while we observed decreased proliferation in CD8^+^ TILs from LL2/B3a tumors (Figure 4G). The impaired proliferation and decreased IFN-γ and TNF-α production might suggest an exhausted phenotype. Indeed, we observed increased expression of PD-1 and CTLA-4 in LL2/B3a-derived CD8^+^ TILs compared with LL2/Ctrl-derived CD8^+^ TILs. Radiation further promoted CTLA-4 expression in LL2/B3a-derived CD8^+^ TILs, indicating increased T cell exhaustion in LL2/B3a tumors (Figure 4H).

### Depletion of CD11b^+^ myeloid cells in SERPINB3 tumors improves T cell activity.

**Figure 5:**
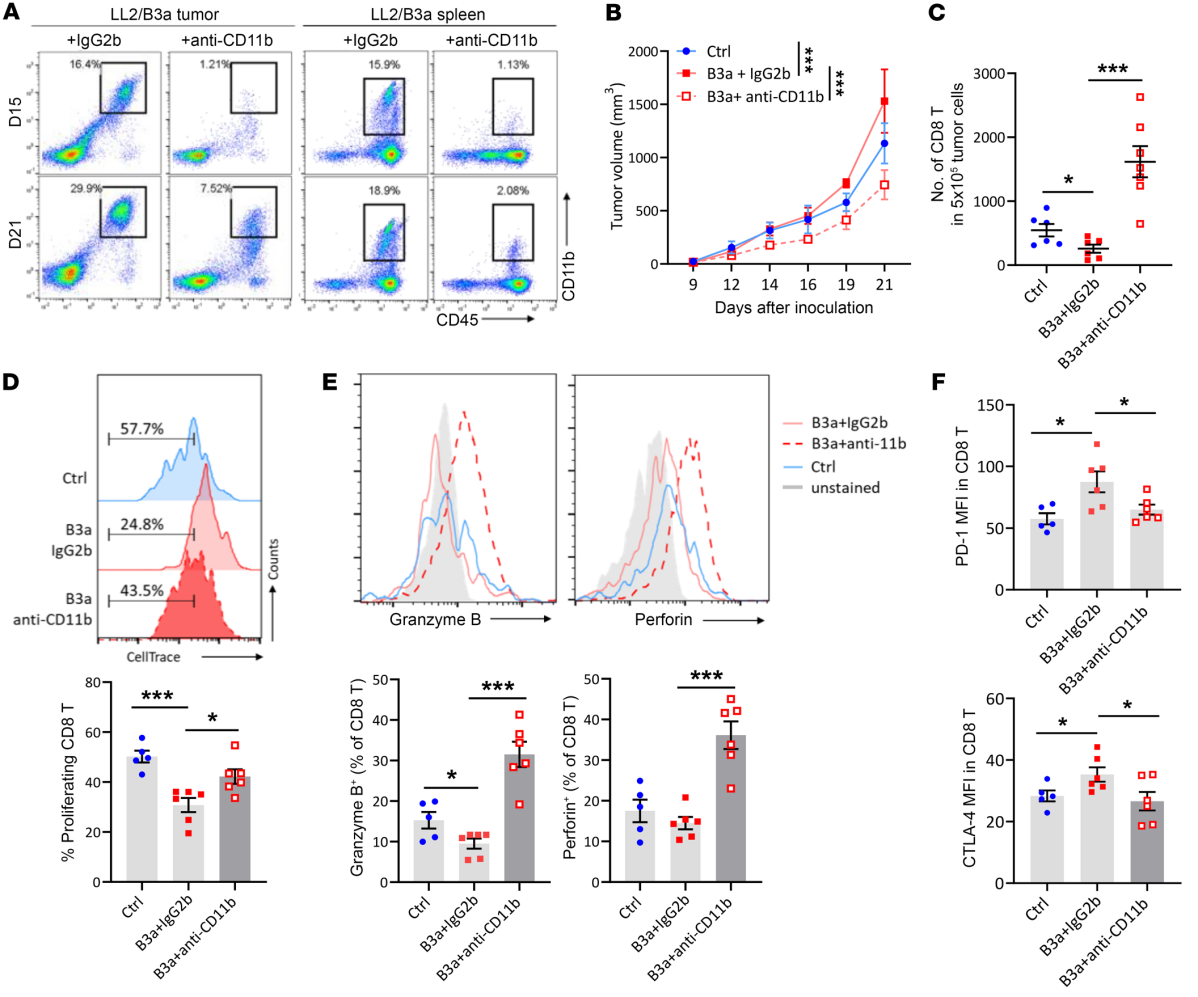
Depleting CD11b^+^ myeloid cells in SERPINB3 tumors improves T cell activity. (**A**) Representative plots show the depletion of CD11b^+^ cells, gated on CD45^+^CD11b^+^ cells, in tumors and spleens on day 15 and day 21 after tumor inoculation. (**B**) Tumor growth of LL2/Ctrl tumors (blue line) and LL2/B3a tumors treated with anti-CD11b antibody (red dotted line) or anti-IgG2b antibody (red solid line). ***P < 0.001, by 2-way ANOVA. (**C**) The numbers of infiltrating CD8^+^ T cells in 5 × 10^5^ total tumor cells were determined by flow cytometry. (**D**) CellTrace-labeled intratumoral T cells were stimulated with anti-CD3/anti-CD28 antibody for 4 days, and cell proliferation was determined by the dilution of CellTrace. (**E**) Representative histograms of intracellular cytokine staining of granzyme B and perforin in CD8^+^ T cells. (**F**) PD-1 and CTLA-4 expression was examined by flow cytometry and is shown as MFI. Data in **C**–**F** are shown as the mean ± SEM, and each dot represents a biologically independent sample. *P < 0.05 and ***P < 0.001, by 1-way ANOVA with Tukey’s post hoc test.

To determine whether impaired T cell activity in LL2/B3a tumors was associated with high infiltration of immunosuppressive myeloid cells, we treated LL2/B3a-tumor bearing mice with CD11b-neutralizing antibody to deplete myeloid cells or IgG2b isotype control starting on day 9 after tumor inoculation. Splenic and intratumoral depletion of CD11b^+^ cells was examined on days 15 and 21, at which point efficient depletion was observed in spleens on both days but slightly recovered in tumors on day 21 (Figure 5A). The growth of LL2/B3a tumors was significantly reduced by anti-CD11b antibody treatment compared with LL2/B3a treated with IgG2b control or LL2/Ctrl tumors (Figure 5B). A decreased total number of CD8^+^ T cells in LL2/B3a tumors compared with LL2/Ctrl tumors was reversed by the depletion of CD11b^+^ cells (Figure 5C). This also relieved the suppression of CD8^+^ T cells to enhance their activity in LL2/B3a tumors, where a lower response of CD8^+^ T cells to CD3/28-induced activation was increased in anti-CD11b–treated LL2/B3a tumors (Figure 5D). The expression of cytotoxic granules, perforin, and granzyme B was significantly increased in CD8^+^ T cells from anti-CD11b–treated LL2/B3a tumors compared with IgG2b-treated LL2/B3a and LL2/Ctrl tumors (Figure 5E). The improved T cell activity in anti-CD11b–treated LL2/B3a tumors was accompanied by reduced PD-1 and CTLA-4 expression, which was highly expressed in IgG2b-treated LL2/B3a tumors (Figure 5F).

High numbers of myeloid cells in LL2/B3a tumors can be a therapeutic target to restore the T cell antitumor response; however, clinical trials targeting myeloid cell integrins such as CD11b/CD18 have failed to yield therapeutic benefits, given the limitation of tolerable doses in human (23, 24). We also found that even though tumor sizes were smaller with anti-CD11b antibody treatment, the tumor doubling time remained the same from days 14–21, suggesting that once tumors were established, the growth of tumor cells was not inhibited by CD11b^+^ cell depletion (Supplemental Figure 7). This may be in part due to a multifaceted role of CXCL1 and S100A8/A9 secreted by LL2/B3a tumors in promoting tumor cell proliferation and survival (25). Therefore, targeting SERPINB3 may be an alternative approach to reduce tumor growth and provide therapeutic potential.

### Targeting SERPINB3 sensitizes tumors to RT and enhances the T cell response.

**Figure 6:**
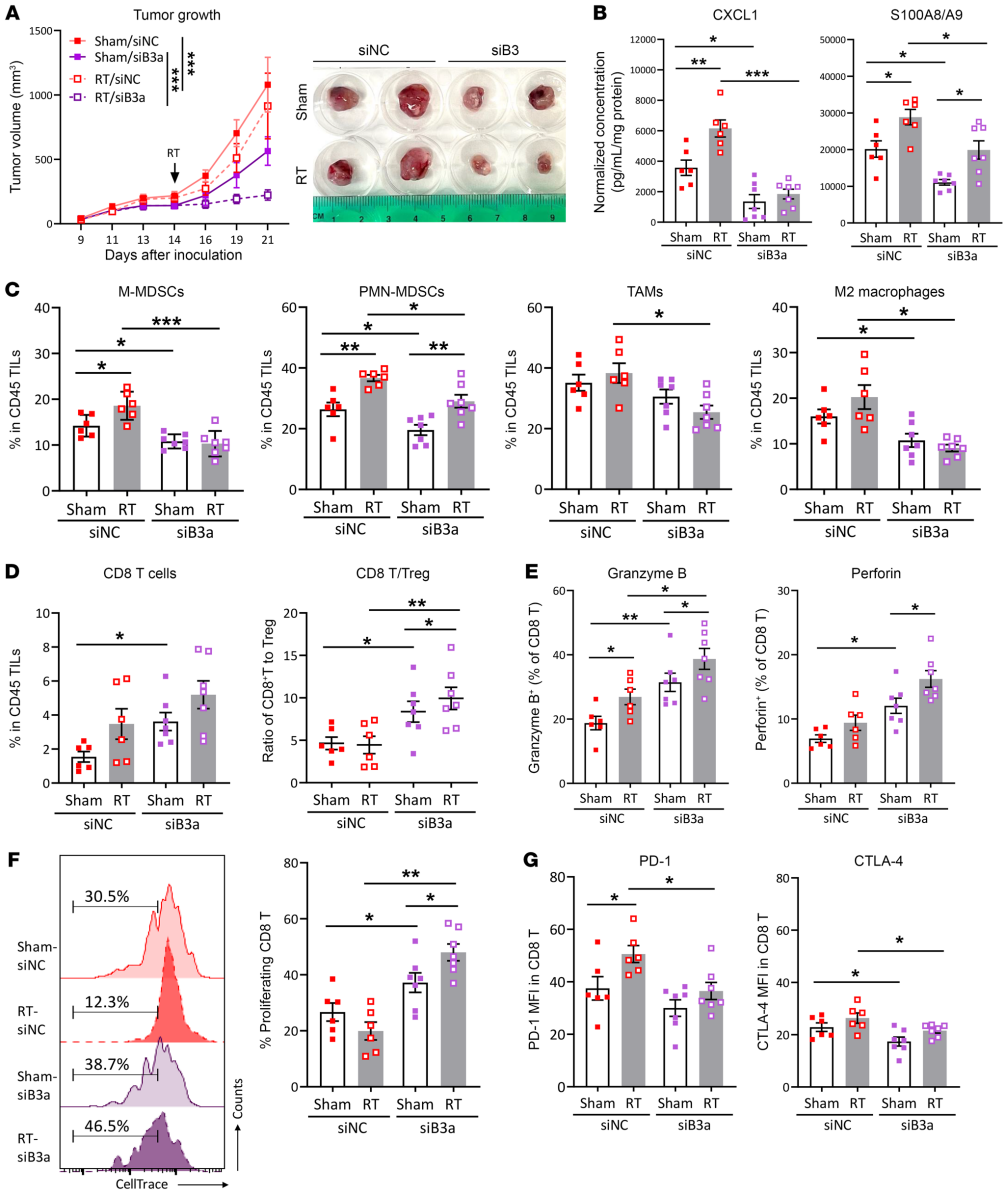
Targeting SERPINB3 sensitizes tumors to RT and enhances T cell response. (**A**) Growth curves of LL2/B3 tumors treated with siNC (red lines) and siB3 (purple lines) with or without RT (sham: solid lines; RT: dotted lines). ***P < 0.001, by 2-way ANOVA. (**B**) The chemokines CXCL1 and S100A8/A9 in tumor homogenates were assessed by ELISA. Data were normalized to the protein concentration for each tumor homogenate. (**C** and **D**) Cumulative data from FACS analysis show the (**C**) frequencies of immune cell populations including CD11b^+^Ly6G-Ly6C^hi^ M-MDSCs, CD11b^+^Ly6G^+^ PMN-MDSCs, CD11b^+^Ly6G-F4/80^+^ TAMs, and CD11b^+^Ly6G-F4/80^+^CD163^+^ M2 macrophages, as well as (**D**) CD3^+^CD8^+^ T cells in total TILs and the ratio of CD3^+^CD8^+^ T cells to CD4^+^CD25^+^Foxp3^+^ Tregs. (**E**) Intracellular cytokine staining for granzyme B and perforin in CD8^+^ T cells was analyzed by flow cytometry. (**F**) CellTrace-labeled intratumoral T cells were stimulated with anti-CD3/anti-CD28 antibody for 4 days, and cell proliferation was determined by the dilution of CellTrace. (**G**) The expression of PD-1 and CTLA-4 was examined by flow cytometry and is shown as MFI. Data in **B**–**G** are shown as the mean ± SEM, and each dot represents a biologically independent sample. *P < 0.05, **P < 0.01, and ***P < 0.001, by 1-way ANOVA with Tukey’s post hoc test.

We sought to understand the potential of targeting SERPINB3 in tumor growth inhibition and whether combination with RT could enhance antitumor immunity. To this end, we treated LL2/B3a tumors with *Serpinb3a* siRNA (siB3) or negative control siRNA (siNC) on day 9 after tumor inoculation with repeated injection every 2–3 days, and a single dose of 10 Gy or sham RT was given on day 14. KD of *Serpinb3a* showed an average of decrease of 65% in siB3-treated tumors (Supplemental Figure 8A). We observed reduced tumor growth in sham siB3–treated tumors compared with sham siNC–treated tumors, and the combination of *Serpinb3a* KD with RT (RT/siB3) resulted in more significant tumor growth inhibition (Figure 6A and Supplemental Figure 8B). We observed a significant decrease in the high expression of CXCL1 and S100A8/A9 in LL2/B3a tumors following *Serpinb3a* KD (sham/siNC vs. sham/siB3). RT induced S100A8/A9 in both RT/siNC and RT/siB3 tumors but not CXCL1, which was induced only in RT/siNC tumors compared with their sham counterparts (Figure 6B). Reduced suppressive chemokine secretions in siB3 tumors also led to decreased myeloid cell infiltration, in which a significant reduction in M-MDSCs, PMN-MDSCs, and M2 macrophages in sham/siB3 tumors was observed. We detected an increase in PMN-MDSCs after radiation in both RT/siNC and RT/siB3 tumors, whereas an increase in M-MDSCs by RT was only seen in RT/siNC, but not RT/siB3, tumors (Figure 6C). In addition, radiation-induced antitumor immunity relies on cytotoxic T cell infiltration. We observed an increase in CD8^+^ T cells and CD8^+^ T cell/Treg ratios in sham/siB3 tumors versus sham/siNC tumors as well as higher ratios of CD8^+^ T cells/Tregs in RT/siB3 versus sham/siB3 tumors, suggesting that Serpinb3a KD increased CD8^+^ T cell infiltration and reduced Treg infiltration and that RT-induced CD8^+^ T cell infiltration was not accompanied by significant Treg expansion in *Serpinb3a*-KD tumors (Figure 6D). CD8^+^ T cells in siB3 tumors also demonstrated improved cytotoxic potential with increased expression of granzyme B and perforin, which was further enhanced by RT (Figure 6E). Similarly, we observed improved T cell activity upon anti-CD3/anti-CD28 stimulation in siB3 tumor–derived CD8^+^ T cells, which showed a markedly higher proliferative capacity than did those derived from siNC tumors (Figure 6F). This correlated with lower expression of PD-1 in RT/siB3 versus RT/siNC tumors and of CTLA-4 in sham/RT- siB3 versus siNC tumors, indicating less exhausted CD8^+^ T cells with silencing of *Serpinb3a* in tumors (Figure 6G). Collectively, targeting *Serpinb3a* resulted in the remolding of infiltrating myeloid cells and a reduction of immunosuppressive chemokines, together with enhanced T cell function, and combining *Serpinb3a* KD with RT achieved more significant inhibition of tumor progression and improved radiation-induced antitumor immunity.

### SERPINB3 mediates suppressive chemokine production through the promotion of STAT activation.

**Figure 7:**
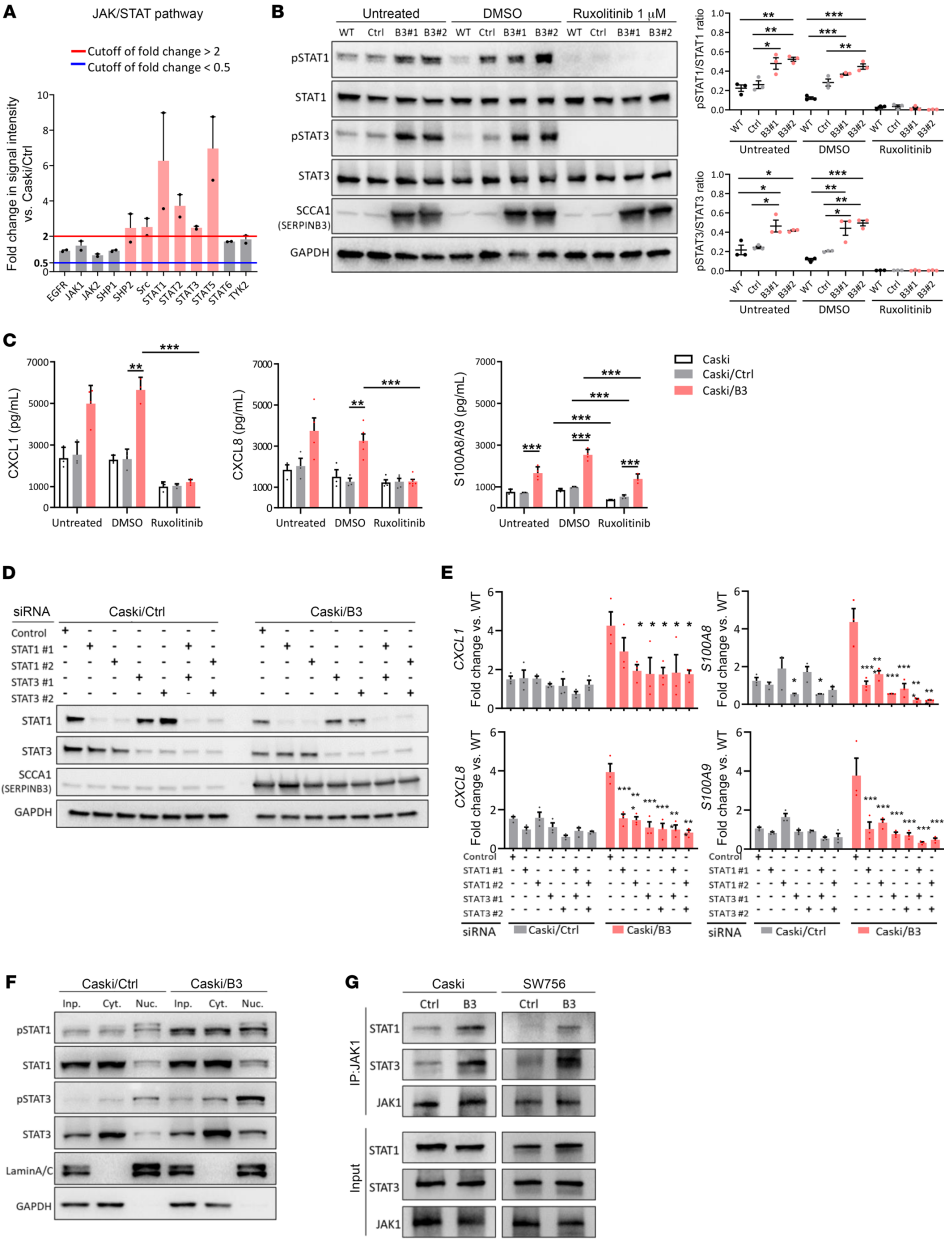
SERPINB3 mediates suppressive chemokine production by promoting STAT activation. (**A**) Activation of JAK/STAT pathway-associated proteins was evaluated by phosphorylation antibody array. Fold changes in phosphorylation were calculated by normalizing the intensity to the expression levels in Caski parental cells and comparing the phosphorylation intensity in Caski/B3 cells with the levels in Caski/Ctrl cells. The red line indicates a fold change of 2 or greater, and the blue line indicates a fold change of 0.5 or less. (**B**) Immunoblotting (left) and quantification (right) show the inhibition of STAT1/3 phosphorylation after treating Caski parental cells (WT), Caski/Ctrl cells (**C**), and Caski/B3 (B3#1, B3#2) cells with 1 μM ruxolitinib for 48 hours. (**C**) Caski/WT, Caski/Ctrl, and Caski/B3 cells were treated with 1 μM ruxolitinib, and the secretion of CXCL1, CXCL8, and S100A8/A9 was assessed by ELISA. (**D**) Immunoblotting shows the KD of STAT1/3 by siRNA in Caski cells. (**E**) The expression of *CXCL1/8* and *S100A8/A9* mRNA was examined by qPCR. Gene expression was normalized to *GAPDH*. Fold changes and significance were calculated by comparing to the expression levels in Caski/Ctrl cells transfected with the negative control siRNA. (**F**) p-STAT1/3 expression in the nucleus (Nuc.), cytoplasm (Cyt.), and total cell lysates (input, Inp.) was measured by immunoblotting. (**G**) Immunoprecipitation using anti-JAK1 antibody shows increased interaction with STAT1 and STAT3 in Caski/B3 and SW756/B3 cells compared with Caski/Ctrl and SW756/Ctrl cells, respectively. Data are shown as the mean ± SEM of 3 experiments. *P < 0.05, **P < 0.01, and ***P < 0.001, by 1-way ANOVA with Tukey’s post hoc test.

Although SERPINB3 has been implicated in proinflammatory signaling in pancreatic cancer and Kras-mutant tumors (26), the underlying molecular mechanism is unknown. To provide further insight into the SERPINB3-mediated suppressive immune response, we used a human phosphorylation pathway profiling array that contained 5 cancer-associated pathways — MAPK, AKT, JAK/STAT, NF-κB, and TGF-β — and identified 14 proteins with upregulated phosphorylation (fold change ≥2) and 4 proteins with downregulated phosphorylation (fold change ≤ 0.5) in Caski/B3 versus Caski/Ctrl cells (Supplemental Figure 9). Among those with increased phosphorylation, STATs proteins, including STAT1/-2/-3/-5, showed the highest magnitude of change in phosphorylation (Figure 7A). Phosphorylation of STAT1 and STAT3 in response to SERPINB3 expression was examined in Caski and SW756 cells, where the induction of SERPINB3 resulted in increased p-STAT1 and p-STAT3 (untreated, Figure 7B and Supplemental Figure 10A). In contrast, KD of SERPINB3 led to reduced p-STAT1 and p-STAT3 expression (Supplemental Figure 10B). Thus, we hypothesized that SERPINB3 mediates suppressive chemokine production by promoting the activation of STAT signaling. The FDA-approved small-molecule inhibitor ruxolitinib inhibition of STAT1 and STAT3 phosphorylation was confirmed by immunoblotting (Figure 7B and Supplemental Figure 10A). The initially high secretion of CXCL1/8 and S100A8/A9 in Caski/B3 and SW756/B3 cells was significantly suppressed by ruxolitinib, suggesting an essential role of STAT activation in chemokine production in SERPINB3 cells (Figure 7C and Supplemental Figure 10C). To further understand whether STAT signaling directly regulates chemokine expression in SERPINB3 cells, we used an siRNA to individually silence STAT1 or STAT3 (Figure 7D and Supplemental Figure 10D). The expression of CXCL1/-8 and S100A8/A9 was decreased in SERPINB3 cells by silencing either STAT1 or STAT3, and the simultaneous KD of both STAT1 and STAT3 did not lead to more significant suppression of CXCL1 and CXCL8. However, KD of both STAT1 and STAT3 achieved more effective inhibition of S100A8 and S100A9 in SERPINB3 cells (Figure 7E and Supplemental Figure 10E). Moreover, both STAT1 and STAT3 proteins showed increased phosphorylation and nuclear translocation in SERPINB3 cells, indicating upregulated transcriptional activity in promoting downstream gene expression (Figure 7F). Notably, an increase in p-STAT1/3 was not only observed in the nucleus but also the cytoplasm, which suggests that SERPINB3 may be involved in mediating upstream cytoplasmic kinase of the signaling cascade to promote STAT activation. Thus, we performed co-immunoprecipitation of JAK1 and found increased interaction of JAK1 with STAT1 and STAT3 in Caski/B3 and SW756/B3 cells compared with Caski/Ctrl and SW756/Ctrl cells (Figure 7G). These data show that SERPINB3 mediated STAT activation by promoting JAK/STAT interaction, leading to increased STAT transcriptional activity and chemokine production.

### Elevated serum SCCA and high tumor p-STAT3 are associated with CD11b expression and poor cancer-specific survival after CRT.

**Figure 8:**
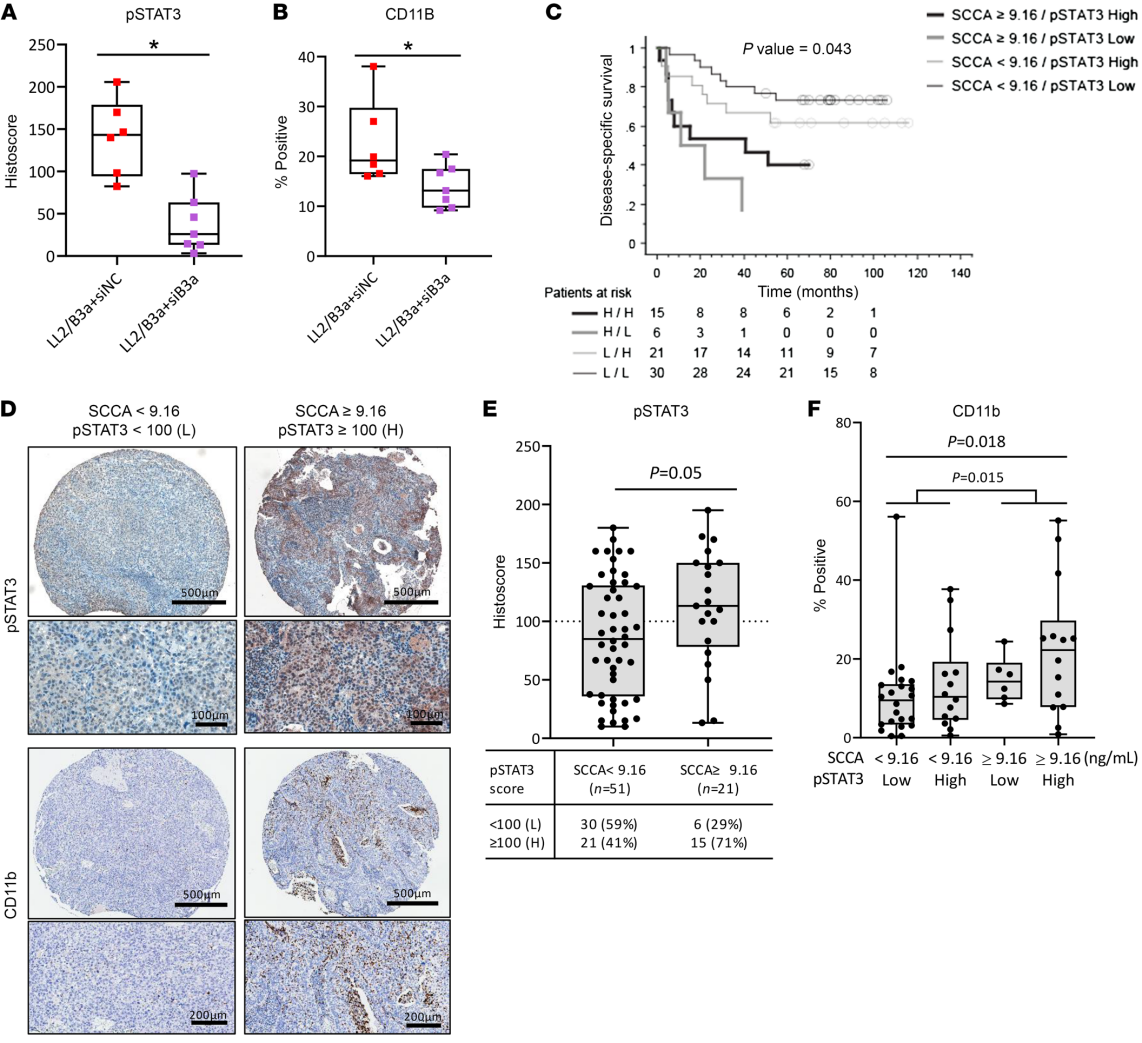
Elevated serum SCCA levels and high tumor p-STAT3 are associated with CD11b expression and poor cancer-specific survival after CRT. (**A** and **B**) Quantification of immunostaining for p-STAT3 and CD11b expression in mouse tumors treated with siNC or siB3. Box plots show p-STAT3 staining scores and the percentage of CD11b^+^ staining from 8–12 representative fields each for 6–7 mice per group. Box plot whiskers span the minimum and maximum values; lines represent the median. (**C**) Kaplan-Meier plot shows overall survival for patients with serum SCCA levels below 9.16 ng/mL and a p-STAT3 histoscore below 100 (*n* = 30) or of 100 or higher (*n* = 21), compared with patients with serum SCCA levels of 9.16 ng/mL or higher with a p-STAT3 histoscore below 100 (*n* = 6) or of 100 or higher (*n* = 15). The average pretreatment serum SCCA value of 9.16 ng/mL from 72 patients with cancer was used as a cutoff. L, low; H, high. (**D**) Representative images of p-STAT3 and CD11b staining for patients with SCCA levels below 9.16 ng/mL or of 9.16 ng/mL or higher. Scale bars: 100 μm, 200 μm, and 500 μm. (**E**) p-STAT3 staining score (histoscore) for patients with serum SCCA levels below 9.16 ng/mL versus those with SCCA levels of 9.16 ng/mL or higher. (**F**) Percentage of the myeloid cell marker CD11b staining in patients with serum SCCA levels below 9.16 ng/mL or of 9.16 ng/mL or higher and a p-STAT3 histoscore below 100 (low) or of 100 or higher (high). Each dot represents an individual patient. Data are shown as the mean ± SEM. A Mann-Whitney *U* test was used to determine statistical significance.

In agreement with our in vitro findings, mouse LL2/B3a tumors with initially high p-STAT3 expression were significantly reduced by intratumoral KD of *Serpinb3a* (LL2/B3a plus siB3), as evidenced by immunostaining for p-STAT3(Tyr705) (Figure 8A and Supplemental Figure 11A). The reduction of p-STAT3 in *Serpinb3a*-KD tumors further correlated with reduced CD11b^+^ myeloid cell expression (Figure 8B and Supplemental Figure 11A).

To evaluate the clinical implication of our findings, tissue microarrays containing pretreatment cervix tumor biopsy specimens obtained from patients with biopsy-proven invasive cervical carcinoma were immunostained for p-STAT3(Tyr705) and the myeloid cell marker CD11b. The average of pretreatment SCCA values from 72 patients (9.16 ng/mL) presented a significant cutoff point for cancer-specific survival in our patient population. Patients with elevated pretreatment SCCA levels (≥9.16 ng/mL) had worse survival than did those with low SCCA levels at the time of diagnosis (Figure 8C and Supplemental Figure 11B), in agreement with our previous study reporting SCCA as a clinical biomarker. The histoscore of p-STAT3 evaluated by IHC was determined through the combined factors of the intensity and percentage of stained cells within the tumor proportion of the tissue microarray (TMA) cores using an attribute cutoff of 100 for high or low p-STAT3 expression (Figure 8D). In the high pretreatment SCCA patient cohort (≥9.16 ng/mL), 71% of the cohort had a high p-STAT3 histoscore as opposed to 41% of the patients with low pretreatment serum SCCA levels (<9.16 ng/mL) (Figure 8E). Although p-STAT3 was not an independent prognostic factor for survival in our patient cohort, elevated serum SCCA levels, along with high p-STAT3, were associated with increased CD11b expression (Figure 8F) and poor cancer-specific survival on both univariate and multivariate analyses, along with International Federation of Gynecology and Obstetrics (FIGO) stage (Figure 8C and Supplemental Table 2). In contrast, the majority of patients with pretreatment serum SCCA levels below 9.16 ng/mL had a low p-STAT3 histoscore and low B3, both of which correlated with low CD11b expression. This cohort had the highest cancer-specific survival rates (Figure 8C). Overall, SCCA is a strong clinical biomarker, and when combined with p-STAT3 expression, it may indicate an unfavorable TME and provide an opportunity for the selection of patients for anti-STAT– and/or anti-SERPINB3–directed therapies.

## Discussion

In this study, we revealed that SERPINB3 modulated the TME toward an immunosuppressive phenotype by upregulating CXCL1/8 and S100A8/A9 production to facilitate tumor growth and impede the success of RT. These chemokines were increased in the tumors of both human SERPINB3-expressing Caski xenografts and murine Serpinb3a-expressing LL2 syngeneic mouse models, resulting in increased infiltrating M-MDSCs, PMN-MDSCs, and M2 macrophages. Radiation-induced T cell responses were compromised by the suppressive microenvironment in SERPINB3 tumors. In contrast, targeting SERPINB3 in tumors reversed these effects on the TME by reducing immunosuppressive chemokine production and myeloid cell infiltration, which led to enhanced T cell activity. More important, targeting SERPINB3 in combination with RT showed significant tumor growth inhibition and improved RT-induced T cell immunity. It is worth noting that the correlation between SERPINB3 and CXCL1/8, S100A8/A9 was conserved across several cancers known to have high SERPINB3 expression and that are often associated with poor treatment outcomes, suggesting wide application of our study to a variety of tumors with SERPINB3 expression.

The association between SERPINB3 and chemokines has been reported in atopic dermatitis and psoriasis, in which downregulation of SERPINB3 in keratinocytes was associated with reduced expression of CXCL1/-5/-8 (27) and S100A8 (16). Catanzaro and colleagues showed that SERPINB3 is a downstream mediator of mutant Ras–induced tumorigenesis and that KD of SERPINB3 led to decreased production of IL-6, CXCL1, and CXCL8, thereby suppressing tumorigenesis (26). In patients with cervical cancer or esophageal squamous cell carcinoma, high expression of SERPINB3 was associated with lymph node metastasis (28–30); however, the underlying causes are unknown. Given the crosstalk between chemokines and immune cells, we revealed that the secretion of CXCL1/8 and S100A8/A9 by SERPINB3-expressing tumors resulted in increased immunosuppressive myeloid cell infiltration, and these cell populations have been shown to promote tumor progression by disrupting T cell activation signaling (31), facilitating tumor angiogenesis (32), and activating neoplastic cell invasion and formation of a premetastatic niche (33). The increased immunosuppressive myeloid cell populations provide a possible mechanism for the high metastatic tendency of SERPINB3 tumors and represent a potential target for tumor control.

Strategies for targeting myeloid cells to improve T cell–mediated immunity or alter myeloid cell polarization and infiltration have been studied extensively in the preclinical space. For instance, CXCR1/2 and CCR2 inhibitors interrupted the CXCL8/CXCR1-2 and CCL2/CCR2 axis, blocking the recruitment of TAMs and MDSCs (34). The CSF-1 receptor inhibitor repolarized TAMs from a M2-like to a M1-like phenotype and depleted TAMs to reduce suppressive immune responses, but studies also found increased PMN-MDSC infiltration through a CXCR2-dependent mechanism (35–37). Other compensatory actions, such as the expansion of monocytes and macrophages when targeting granulocytes and compensatory upregulation of PD-L1 and CTLA-4 by untargeted myeloid cells have also been reported (38, 39). This limits the therapeutic efficacy of myeloid cell–targeting strategies. Similarly, when we depleted CD11b^+^ myeloid cells in SERPINB3 tumors, we observed initial tumor growth inhibition and improved T cell activity. However, after the initial tumor growth delay, we noticed no difference in the tumor doubling time between CD11b-depleted and -nondepleted tumors, and an immune cell population with intermediate levels of CD11b expression appeared in the CD11b-depleted tumors. This suggests that combination therapy or additional strategies to overcome compensatory mechanisms triggered by myeloid cell–targeting therapies might be required.

Hyperactivated STAT3 signaling has been shown to mediate immunosuppression through tumor cell–intrinsic and –extrinsic mechanisms and is associated with poor clinical prognosis for many cancers, including cervical, lung, and head and neck cancers (40–42), in which elevated SERPINB3 expression is also frequently observed. Increased STAT3 activity was found to inhibit the production of immunogenic cytokines/chemokines, induce the expression of PD-1/PD-L1, and regulate suppressive immune activities in Tregs and MDSCs (43–45). Therefore, the potential of inhibiting STAT activity to improve therapeutic responses has been explored in many preclinical studies. Ruxolitinib, the first FDA-approved JAK/STAT pathway inhibitor, successfully triggered tumor regression in preclinical mouse models; however, clinical trials of pancreatic adenocarcinoma (46), breast cancer (47), colorectal cancer (48), and lung cancer (49) showed s lack of efficacy and very limited or no overall survival benefit. Among a few other ongoing trials, a phase I study of glioblastoma (NCT03514069) showed a promising preliminary result from combing ruxolitinib with radiation and temozolomide (50). A recently completed trial (NCT01904123) used the STAT inhibitor WP1066 in combination with radiation to treat patients with recurrent malignant glioma. Their preclinical study showed that the STAT3 inhibitor and irradiation reprogrammed the immunosuppressive glioma TME by improving DC maturation and interactions with T cells (51). Of note, HPV-related cancers, including cervical and head and neck cancers, often showed hyperactivated STAT3 due to virus-associated inflammatory responses (52). Ruxolitinib demonstrated in vitro effects by facilitating cisplatin-induced cell death in HPV^+^ cervical cancer cells (53); however, in vivo efficacy has not been investigated, and whether this success can transition to clinical trials remains unclear.

In addition, knowing that STAT transcriptional activity is also involved in T cell function (54) and other facets of the immune response, direct inhibition of this pathway may unintentionally tip the immune axis back in favor of the tumor. Here, we provide the rationale for targeting an upstream, tumor-specific signal — SERPINB3 — as a more effective approach in the clinical setting. Silencing SERPINB3 led to a reduction in infiltrating immunosuppressive myeloid cells and in return enhanced T cell responses. The changes in the TME further rendered RT effective in previously radioresistant SERPINB3 tumors. In addition to affecting myeloid-derived cell recruitment, SERPINB3 silencing also abrogated CTLA4 expression. The potential connection between SERPINB3 and immune checkpoints has also been reported in HPV^–^ head and neck squamous cell carcinoma, in which high SERPINB3 expression in patients corresponded to increased PD-L1 and PD-L2 (55). Similarly, genome-level and IHC analyses showed upregulated PD-L1 in SERPINB3-high ovarian and esophageal tumors (56). Although SERPINB3 KD improved cytotoxic T cell function, an increase in PMN-MDSCs and slightly higher PD-1 expression were observed following RT. Therefore, a combined therapy of targeting SERPINB3 and immune checkpoint inhibitors may simultaneously reduce immunosuppressive chemokine–associated induction of myeloid cells and prevent T cell exclusion and dysfunction, leading to maximal RT-induced antitumor immunity. The HPV subtype or positivity did not appear to be associated with SERPINB3 expression in our patient cohort, thus this may be a viable approach for patients with HPV- or non–HPV-associated cervical cancer.

Our findings that SERPINB3 modulated the crosstalk between immune cells and cancer cells via secretion of CXCL1/8 and S100A8/A9 implicate this protease inhibitor member of the SERPIN superfamily in a key tumor strategy of evading antitumor immune responses and resisting therapies such as radiation. This study implicates SERPINB3 in the promotion of interaction with and activation of STATs by upstream kinases, and the direct molecular mechanism is major focus of ongoing studies. Targeting SERPINB3 reprogrammed the immunosuppressive environment and sensitized the tumor to RT. Our study also presents several potential therapeutic combinations, such as the use of RT with STAT inhibitors or with immune checkpoint blockade, to further improve treatment responses for cancers with elevated SERPINB3 expression.

## Methods

### Cell lines and plasmids.

Caski, SW756, and C33a cells were purchased from the American Type Culture Collection (ATCC) and Lewis lung carcinoma (LL2/LLC) cells were a gift of Dinesh Thotala (Washington University School of Medicine, St Louis, Missouri, USA). Cells were cultured in IMDM or DMEM, supplemented with 10% FBS and 100 U/mL penicillin-streptomycin. SERPINB3 CRISPR/Cas9-KO cell generation was described previously (10). Cells stably expressing SERPINB3 were generated using the pULTRA lentiviral vector (Addgene no. 24129) containing human SERPINB3 or pLV-C-GFPSpark vector (Sino Biological LVCV-35) containing mouse Serpinb3a GFP-tagged fusion proteins. SERPINB3-KD cells were transduced with scrambled shRNA (Addgene no. 1684) or SERPINB3 shRNA (Sigma Mission shRNA, TRCN0000052400). Genetically modified cells were generated through a lentivirus system by transfection of human 293T packaging cells. All cell lines were grown in a monolayer at 37°C with 5% CO_2_ and periodically tested for *Mycoplasma* contamination.

### RNA-Seq and TCGA data analysis.

RNA-Seq was performed on pretreatment tumor biopsies obtained from patients enrolled in a prospective tumor banking study. Tumor samples with extracted RNA exceeding thresholds for quality and quantity as defined by TCGA were submitted for whole-transcriptome sequencing (*n* = 66). PolyA selection was performed before multiplexed sequencing (Illumina HiSeq 3000, 1 × 50 nt, ~40 million reads per sample). Sequenced reads were aligned to the human reference genome GRCh38 (r90) using STAR, version 2.7.0f, and aligned reads were sorted and indexed using sambamba, version 0.6.9. Gene expression was quantified using featureCounts, version 1.6.4, to obtain read counts and cufflinks, version 2.2.1, to obtain normalized fragments per kilobase per million mapped reads (FPKM). In downstream analyses, genes with consistently low expression (i.e., <1 FPKM or <200 reads) in at least 95% of the samples were excluded, as reported previously (57). Raw sequencing reads and expression data are available in the NCBI’s Gene Expression Omnibus (GEO) database (GEO GSE151666). TCGA RNA-Seq data were obtained through cBioPortal (https://www.cbioportal.org/). Correlations between SERPINB3 expression and that of other genes were evaluated using Spearman’s correlation coefficient. *P* values were adjusted for multiple testing by the method of Benjamini and Hochberg, with an adjusted *P* value of less than 0.05 considered to be significant. Immune cell populations and enrichment scores were analyzed using xCell analysis, a gene signature–based method to estimate cell composition in bulk transcriptomic data (20). For correlation analysis and enriched immune cell gene signatures, *P* values were corrected with a FDR of less than 0.05. Heatmaps were generated using GraphPad Prism (GraphPad Software) and are based on the average score for each immune cell subtype in our predefined patient groups.

### PBMC isolation and Transwell assays.

Fresh primary PBMCs were obtained from patients planning to undergo brachytherapy RT for cervical cancer and had enrolled under a prospective biospecimen banking protocol. Fresh blood was collected in EDTA separator tubes, and PBMCs were immediately isolated using Lymphoprep and SepMate-50 (STEMCELL Technologies) centrifugation tubes, according to the manufacture’s instructions. Transwell assays were performed using 8 μm Transwells (Falcon). Supernatants were collected from cells cultured in complete growth media for 48 hours and loaded into the lower chamber of the Transwell. PBMCs were loaded into the upper Transwell for a 4-hour migration period. Migrated cells were phenotyped by flow cytometry.

### Flow cytometry and data analysis.

Single-cell suspensions were blocked with either Human TruStain FcX Solution (422301, BioLegend) or mouse TruStain FcX PLUS (anti–mouse CD16/CD32) antibody (S17011E, BioLegend) to avoid nonspecific Fc receptor binding and stained with a LIVE/DEAD Fixable Dead Cell Stain Kit (MACS) to exclude dead cells. For surface staining, cells were incubated with the appropriate antibodies for 30 minutes at 4°C. Intracellular cytokine and nuclear staining was performed after surface staining using the Cyto-Fast Fix/Perm Buffer Set and the True-Nuclear Transcription Factor Buffer Set, respectively (BioLegend). Stained cells were analyzed using a MACSQuant Analyzer 10 Flow Cytometer (Miltenyi Biotec). Antibody details are provided in Supplemental Table 3. Data analysis including viSNE and FlowJo plugin FlowSOM was performed using FlowJo, version 10 (TreeStar). A range of 20,000–60,000 live cells were acquired, and individual flow cytometric data from each group were combined into a single data file to generate viSNE. Color-coded subpopulations were gated by predefined markers for each immune cell type and overlaid on the viSNE plots to show CD45^+^ cells from tumors. All flow cytometric gating plots, histograms, and statistics were generated using FlowJo.

### Mouse tumors with anti-CD11b antibody, siRNA, and/or RT.

For xenograft models, 6- to 7-week-old female athymic nude mice (Charles River Laboratories) were injected subcutaneously in their flank with 5 × 10^6^ Caski/Ctrl or Caski/B3 cells suspended in serum-free IMDM and 50% Matrigel Basement Membrane Matrix (Corning) to a final volume of 100 μL. For immunocompetent models, 7- to 8-week-old female C57/BL6 mice (Charles River Laboratories) were injected subcutaneously into the right flank with 5 × 10^5^ LL2/Ctrl or LL2/B3a cells suspended in 100 μL PBS. To deplete myeloid cells, mice were treated with anti-CD11b antibody (Ultra-LEAF purified anti–mouse CD11b, BioLegend) or isotype IgG2b as a control (Ultra-LEAF purified rat IgG2b, BioLegend). Antibodies were administered through intraperitoneal injection at the initial dose of 300 μg in 150 μL PBS on day 10 after tumor inoculation, and a subsequent dose of 150 μg in 100 μL PBS was given every 3 days. To knock down Serpinb3a, mice received either mSerpinb3a siRNA (5′-ACAUCGAAUUUAACUUCAUtt-3′; 5′-AUGAAGUUAAAUUCGAUGUtt-3′; ID:s73336, Thermo Fisher Scientific) or control siRNA (Ambion in vivo negative control 1, Thermo Fisher Scientific) complexed with Invivofectamine 3 (Thermo Fisher Scientific) as per the manufacturer’s protocol. Mice received 3 intratumoral injections of 10 μg siRNA on days 9, 11, and 13 after tumor inoculation before RT, which was performed on day 14. On days 16 and 19, mice received 20 μg siRNA via intraperitoneal injection. For RT, mice were randomized to receive sham or 10 Gy RT using the Xstrahl Small Animal Radiation Research Platform (SARRP) 200 (Xstrahl Life Sciences). Tumor volume was measured twice weekly and calculated as follows: (length × width^2^)/2. For tissue dissociation, tumors were manually dissected and digested with 1 mg/mL collagenase, 0.5 mg/mL hyaluronidase, and 10 mg/mL DNase I type IV (MilliporeSigma) and then transferred to a tissue disaggregator (Medicon, BD) using CTSV Medimachine II (BD).

### T cell suppression assay.

Intratumoral myeloid cells were isolated from dissociated tumors using the MojoSort mouse CD11b selection kit, biotin anti–mouse Ly6C antibody, biotin anti–mouse Ly6G antibody, biotin anti–mouse F4/80 antibody, and streptavidin nanobeads (BioLegend) through magnetic purification. Splenic T cells were isolated from nontumor-bearing mice and labeled with CellTrace Violet (Thermo Fisher Scientific) to evaluate proliferation. Purified myeloid cells were cocultured with anti-CD3/anti-CD28–activated T cells at a ratio of 1:1 for 4 days. Suppression was determined by CellTrace dilution using FACS and compared with the proliferation of anti-CD3/anti-CD28–activated T cells without myeloid cell coculturing.

### Ex vivo T cell stimulation.

T cells isolated using the MojoSort mouse CD3 T cell isolation kit (BioLegend) were labeled with CellTrace violet and activated with CD3/CD28 Dynabeads (Thermo Fisher Scientific) for 4 days to evaluate proliferation. To examine TNF and IFN, T cells were stimulated with Cell Activation Cocktail (BioLegend) containing 40.5 μM phorbol-12-myristate 13-acetate (PMA) and 669.3 μM ionomycin in the presence of 5 μg/mL brefeldin A for 5 hours and stained with surface and intracellular markers for FACS analysis.

### Serum SCCA and tissue microarray IHC.

Pretreatment serum SCCA levels were evaluated by the ARUP National Reference Laboratory (Salt Lake City, Utah, USA) using ELISA, and the TMA was generated from untreated human tumor specimens, as described previously (14). TMA sections were sent to HistoWiz for IHC staining for CD11b (1:100, Abcam, ab224800), and IHC for p-STAT3 (1:200, MilliporeSigma, SAB4300033) was performed by the Washington University AMP Core Laboratories. Mouse tumor sections were stained with p-STAT3 (1:150, Invitrogen, Thermo Fisher Scientific, PA5-121259) and CD11b (1:500, Invitrogen, Thermo Fisher Scientific, PA5-79532), using the Pierce peroxidase IHC detection kit (Thermo Fisher Scientific). QuPath, version 0.3.2, software was used for automated analysis of surface and cytoplasmic staining to determine the percentage of cells positive for CD11b. IHC for p-STAT3 was evaluated by a board-certified pathologist with gynecologic expertise, and staining scores were calculated for a minimum of 2 tissue cores for each patient (the percentage of positively stained tumor cells × staining intensity ranged from 0 to 3). Values from at least 2 cores from each sample were considered valid, and an average score was taken.

### siRNA KD, RNA extraction, and qPCR.

Lipofectamine RNAiMAX (Thermo Fisher Scientific) was used for STAT1 siRNA (ID: SASI_Hs02_00343387, SASI_Hs01_00098937, MilliporeSigma), STAT3 siRNA (ID: SASI_Hs01_00121206, SASI_Hs01_00061860, MilliporeSigma), and negative control siRNA (SIC001, MilliporeSigma) transfection, according to the manufacturer’s instructions. RNA was isolated using the GenElute Mammalian Total RNA Miniprep Kit (MilliporeSigma) and reverse transcribed to cDNA using the High Capacity cDNA Reverse Transcription Kit (Thermo Fisher Scientific). Quantitative PCR (qPCR) was performed using PowerUp SYBR Green PCR Master Mix (Applied Biosystems) and the Applied Biosystems 7900 Fast real-time PCR system and software. Each sample was analyzed in triplicate, gene expression levels were normalized to *GAPDH*, and fold changes were calculated using the ΔΔCt method. The primer sequences are detailed in Supplemental Table 4.

### ELISA.

Cell culture supernatant was collected 48 hours after fresh media were added to the adherent cells in a monolayer. Quantification of human/mouse chemokines in tissue culture supernatants and tissue homogenates was performed using a commercially available human CXCL1/GRO α, human IL-8/CXCL8, human S100A8/S100A9 heterodimer, mouse CXCL1/KC, and mouse S100A8/S100A9 heterodimer DuoSet ELISA kits from R&D Systems. The mouse GRO γ ELISA Kit was obtained from Abcam. Chemokine concentrations in samples were determined by interpolation from a standard curve.

### Phosphorylation protein array.

The Human Phosphorylation Pathway Profiling Array C55 consisted of the detection of 55 phosphorylated proteins (RayBiotech). The same amount of protein from each sample was used for screening, and assays were performed according to the manufacturer’s instruction. Array blots were scanned with the Bio-Rad ChemiDoc MP imaging system, and images were processed using the Protein Array Analyzer plug-in (http://image.bio.methods.free.fr/ImageJ/?Protein-Array-Analyzer-for-ImageJ.html) of the ImageJ (NIH) program.

### Co-immunoprecipitation and immunoblotting.

Immunoprecipitation of Jak1 was performed using a Pierce co-immunoprecipitation kit (Thermo Fisher Scientific). Cell fractionation was carried out using the NE-PER nuclear and cytoplasmic extraction reagents kit (Thermo Fisher Scientific). The purity of non-nuclear and nuclear fractions was determined using GAPDH and lamin A/C, respectively. For immunoblotting, cells were lysed with RIPA buffer (Cell Signaling Technology) supplemented with proteinase/phosphatase inhibitors (Thermo Fisher Scientific). Protein concentrations were determined using bicinchoninic acid (BCA) (Thermo Fisher Scientific), and proteins were electrophoresed on 4%–20% gradient gels (Bio-Rad), transferred onto a PVDF blot using the Trans-Blot TurboTransfer system (Bio-Rad), and incubated with the antibodies shown in Supplemental Table 3. Chemiluminescence was detected using ECL reagent (Cytiva) and visualized using the Bio-Rad ChemiDoc MP imaging system and Image Labsoftware (Bio-Rad).

### Statistics.

Statistical analyses were performed using GraphPad Prism 8 (GraphPad Software), and all values are reported as the mean ± SEM. A 2-tailed, unpaired *t* test or Mann-Whitney *U* test was used for 2-group comparisons. A 1-way or 2-way ANOVA was used for multiple comparisons, followed by post hoc analysis. *P* values of less than 0.05 were considered statistically significant.

### Study approval.

All experiments were performed in accordance with relevant guidelines and regulations and approved by the Washington University Institutional Biological and Chemical Safety Committee (protocol 12737, version 2.1). All mouse experiments were approved by the IACUC of Washington University (protocol 20-0470). Research in humans was done with informed consent and approved by the IRB of Washington University (protocol 201105374).

### Data availability.

Raw sequencing reads and expression data are available in the NCBI’s Gene Expression Omnibus (GEO) database (GEO GSE151666). RNA-Seq data from TCGA consortium were obtained through cBioPortal (http://www.cbioportal.org/). This study did not generate new analytic code.

## Author contributions

LC and SM conceptualized the study. LC designed and conducted experiments, acquired data, performed formal analysis, and wrote the manuscript. VS, SW, RF and JY conducted experiments and acquired data. MJI, FR, KJ, JZ, and PC contributed to RAN-Seq data curation and analysis. LS interpreted and analyzed histology data. YH and JL assisted with statistical analysis. SG, CJL, CSS, PWG, and JKS provided resources. SM supervised the study, acquired data, performed formal analysis, acquired funding, and wrote the manuscript. All authors reviewed and edited the manuscript.

## Supplementary Material

Supplemental data

## Version 1 06/06/2023

In-Press Preview

## Version 2 08/01/2023

Electronic publication
