# Supplementary material for: SCCA1/SERPINB3 suppresses antitumor immunity and blunts therapy-induced T cell responses via STAT-dependent chemokine production
Source: J Clin Invest. 2023 Aug 1;133(15):e163841. doi: 10.1172/JCI163841 (PMC10378164; doi:10.1172/JCI163841)
Supplement: Supplemental data [file jci-133-163841-s148.pdf]

SCCA1/SERPINB3 suppresses anti-tumor immunity and blunts therapy-induced T cell responses via STAT-dependent chemokine production

Liyun Chen, Victoria Shi, Songyan Wang, Lulu Sun, Rebecca Freeman, Jasmine Yang, Matthew J Inkman, Subhajit Ghosh, Fiona Ruiz, Kay Jayachandran, Yi Huang, Jingqin Luo, Jin Zhang, Pippa Cosper, Clifford J. Luke, Catherine S Spina, Perry W. Grigsby, Julie K. Schwarz, Stephanie Markovina

Supplemental Figures

Supplemental Figure 1

A

SERPINB3	Significant	P Value
Common myeloid progenitor		
low vs. int	No	0.7867
low vs. high	No	0.9826
int vs. high	No	0.77
Myeloid dendritic cell		
low vs. int	No	0.8652
low vs. high	No	0.4333
int vs. high	No	0.3405
Myeloid dendritic cell activated		
low vs. int	No	0.0741
low vs. high	Yes	0.0253
int vs. high	Yes	0.0001
Plasmacytoid dendritic cell		
low vs. int	No	0.3424
low vs. high	Yes	0.0067
int vs. high	No	0.0774
Monocyte progenitor		
low vs. int	No	0.7884
low vs. high	No	0.1243
int vs. high	No	0.2044
Monocyte		
low vs. int	No	0.6591
low vs. high	Yes	0.0206
int vs. high	No	0.0606
Macrophage		
low vs. int	No	0.8877
low vs. high	Yes	0.0194
int vs. high	No	0.0939
Macrophage M1		
low vs. int	No	0.9943
low vs. high	No	0.1478
int vs. high	No	0.1498
Macrophage M2		
low vs. int	No	0.065
low vs. high	Yes	0.0143
int vs. high	Yes	0.0091
Mast cell		
low vs. int	No	0.5707
low vs. high	No	0.2906
int vs. high	No	0.1046
Neutrophil		
low vs. int	No	0.7326
low vs. high	No	0.9332
int vs. high	No	0.6705
Eosinophil		
low vs. int	No	0.9608
low vs. high	No	0.9429
int vs. high	No	0.982

SERPINB3	Significant	P Value
Common lymphoid progenitor		
low vs. int	No	0.7629
low vs. high	No	0.509
int vs. high	No	0.3361
T cell CD4+ memory		
low vs. int	No	0.9422
low vs. high	No	0.0761
int vs. high	No	0.0649
T cell CD4+ naive		
low vs. int	No	0.6046
low vs. high	No	0.4622
int vs. high	No	0.8279
T cell CD4+ (non-regulatory)		
low vs. int	No	0.844
low vs. high	No	0.9448
int vs. high	No	0.8985
T cell regulatory (Tregs)		
low vs. int	No	0.421
low vs. high	Yes	0.0126
int vs. high	No	0.4693
T cell CD4+ central memory		
low vs. int	No	0.9889
low vs. high	No	0.199
int vs. high	No	0.1942
T cell CD4+ effector memory		
low vs. int	No	0.5579
low vs. high	No	0.5985
int vs. high	No	0.266
T cell CD8+ naive		
low vs. int	No	0.5356
low vs. high	No	0.5676
int vs. high	No	0.9617
T cell CD8+		
low vs. int	No	0.8964
low vs. high	Yes	0.0019
int vs. high	Yes	0.0358
T cell CD8+ central memory		
low vs. int	No	0.5493
low vs. high	Yes	0.0031
int vs. high	No	0.0685
T cell CD8+ effector memory		
low vs. int	No	0.3361
low vs. high	No	0.4473
int vs. high	No	0.8398
T cell gamma delta		
low vs. int	No	0.8413
low vs. high	No	0.3951
int vs. high	No	0.2935

SERPINB3	Significant	P Value
T cell CD4+ Th1		
low vs. int	No	0.4074
low vs. high	Yes	0.0045
int vs. high	Yes	0.0209
T cell CD4+ Th2		
low vs. int	No	0.1065
low vs. high	No	0.0955
int vs. high	No	0.1335
T cell NK		
low vs. int	No	0.0644
low vs. high	Yes	0.0019
int vs. high	No	0.4783
NK cell		
low vs. int	No	0.6759
low vs. high	No	0.7338
int vs. high	No	0.9379
B cell		
low vs. int	No	0.0952
low vs. high	No	0.0613
int vs. high	No	0.2501
B cell memory		
low vs. int	No	0.9037
low vs. high	No	0.7763
int vs. high	No	0.6854
B cell naive		
low vs. int	No	0.9961
low vs. high	No	0.9924
int vs. high	No	0.9885
B cell plasma		
low vs. int	No	0.468
low vs. high	No	0.8337
int vs. high	No	0.606
Class-switched memory B cell		
low vs. int	No	0.0836
low vs. high	No	0.2371
int vs. high	No	0.115

SERPINB3 vs overall immune score		
	Non-recurrence (NR)	Recurrence (R)
Pearson r	0.5909	0.2132
P value	0.0048 *	0.3061 ns

Correlation of SERPINB3 with immune cells and chemokines. **(A)** Tables show the comparisons of immune cells in SERPINB3/Low, SERPINB3/Intermediate (int.) and SERPINB3/High. The p-value were corrected from two-way ANOVA with the false discovery rate < 0.05.

Supplemental Figure 1

B

SERPINB3/High vs SERPINB3/Low (p value)		
Myeloid cells	**NR**	**R**
Common myeloid progenitor	0.395	0.176
Myeloid dendritic cell	0.047*	0.196
Myeloid dendritic cell activated	0.417	0.062
Plasmacytoid dendritic cell	0.087	0.428
Monocyte progenitor	0.158	0.276
Monocyte	0.050*	0.643
Macrophage	0.097	0.049*
Macrophage M1	0.085	0.938
Macrophage M2	0.002**	0.324
Mast cell	0.075	0.068
Neutrophil	0.176	0.585
Eosinophil	0.306	0.369

SERPINB3/High vs SERPINB3/Low (p value)		
B cells	**NR**	**R**
B cell	0.738	0.361
B cell memory	0.201	0.476
B cell naive	0.235	0.766
B cell plasma	0.034*	0.792
Class-switched memory B cell	0.492	0.391

SERPINB3/High vs SERPINB3/Low (p value)		
Lymphocytes	**NR**	**R**
Common lymphoid progenitor	0.153	0.400
T cell CD4+ memory	0.312	0.015*
T cell CD4+ naive	0.641	0.248
T cell CD4+ (non-regulatory)	0.353	0.061
T cell regulatory (Tregs)	0.982	0.002**
T cell CD4+ central memory	0.886	0.063
T cell CD4+ effector memory	0.711	0.457
T cell CD8+ naive	0.068	0.562
T cell CD8+	0.050*	0.253
T cell CD8+ central memory	0.036*	0.193
T cell CD8+ effector memory	0.122	0.299
T cell gamma delta	0.035*	0.211
T cell CD4+ Th1	0.413	0.008*
T cell CD4+ Th2	0.126	0.454
T cell NK	0.547	0.011*
NK cell	0.077	0.871

P value shown in green indicates significantly higher in B3/H compared to B3/L

P value shown in red indicates significantly lower in B3/H compared to B3/L

Correlation of SERPINB3 with immune cells and chemokines. **(B)** Tables show the p-value from multiple t-test analysis for heatmaps of immune cell subsets in non-recurrent (NR) and recurrent (R) B3/High vs B3/Low tumors.

Supplemental Figure 1

C

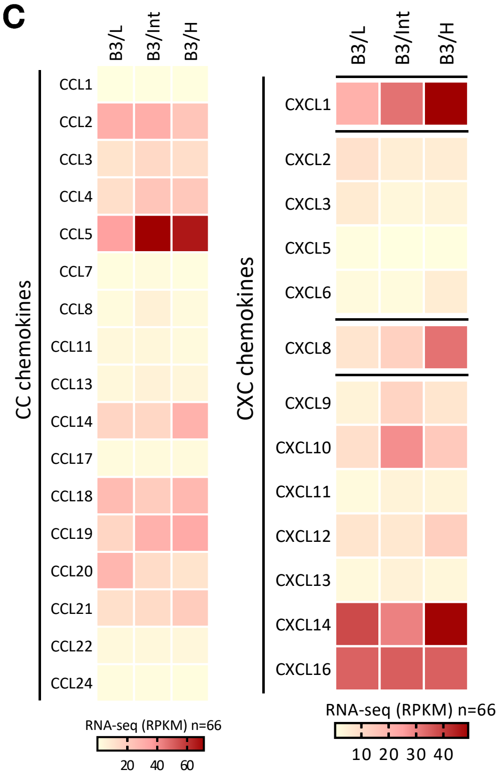

Correlation with SERPINB3	CXCL1	CXCL2	CXCL3	CXCL6	CXCL8	CXCL9	CXCL11	CXCL12
r	0.31	-0.09	-0.04	0.18	0.34	0.16	-0.03	0.03
P value	0.011	0.456	0.738	0.142	0.005	0.193	0.815	0.787
	*	ns	ns	ns	**	ns	ns	ns

Correlation with SERPINB3	CCL1	CCL2	CCL3	CCL4	CCL5	CCL7	CCL8	CCL11	CCL13
r	0.10	-0.06	0.10	0.14	0.06	-0.04	-0.01	-0.11	0.02
P value	0.411	0.609	0.397	0.245	0.632	0.742	0.917	0.381	0.905
	ns	ns	ns	ns	ns	ns	ns	ns	ns

Correlation with SERPINB3	CCL14	CCL17	CCL18	CCL19	CCL20	CCL21	CCL22	CCL24
r	0.22	0.04	0.02	0.05	-0.16	0.17	0.01	-0.12
P value	0.069	0.765	0.844	0.715	0.198	0.168	0.907	0.330
	ns	ns	ns	ns	ns	ns	ns	ns

D

TCGA-CESC

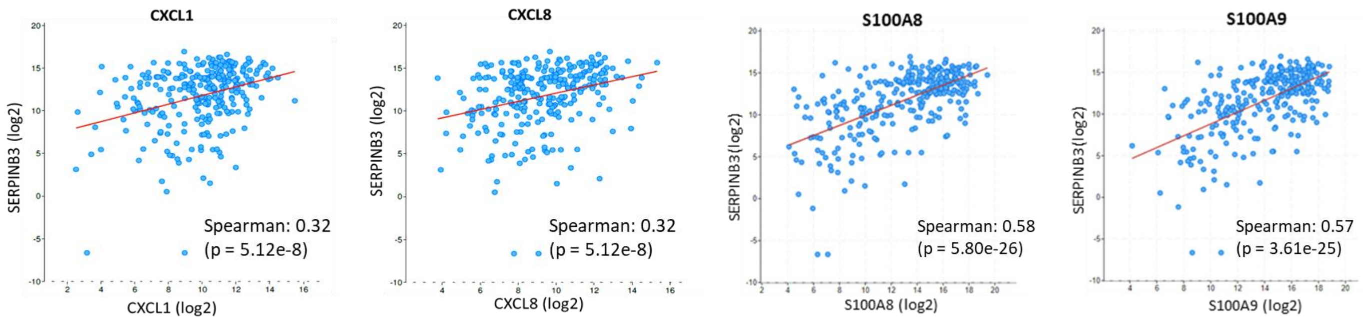

Correlation of SERPINB3 with immune cells and chemokines (C) Heatmaps show the relative transcript levels of CC and CXC chemokines in SERPINB3-low (B3/L, n=22), SERPINB3-intermediate (B3/Int, n=22) and SERPINB3-high (B3/H, n=22) tumors. Color intensity is proportional to average transcript expression across samples in the indicated groups. Table shows the correlation coefficient (r) and significance (p value). **(D)** Correlation of SERPINB3 with the expression of CXCL1, CXCL8, S100A8, S100A9 was analyzed using TCGA-CESE (cervical squamous cell carcinoma and endocervical adenocarcinoma, n=306) RNAseq data. Correlation plots were generated using cBioPortal.

Supplemental Figure 1

E

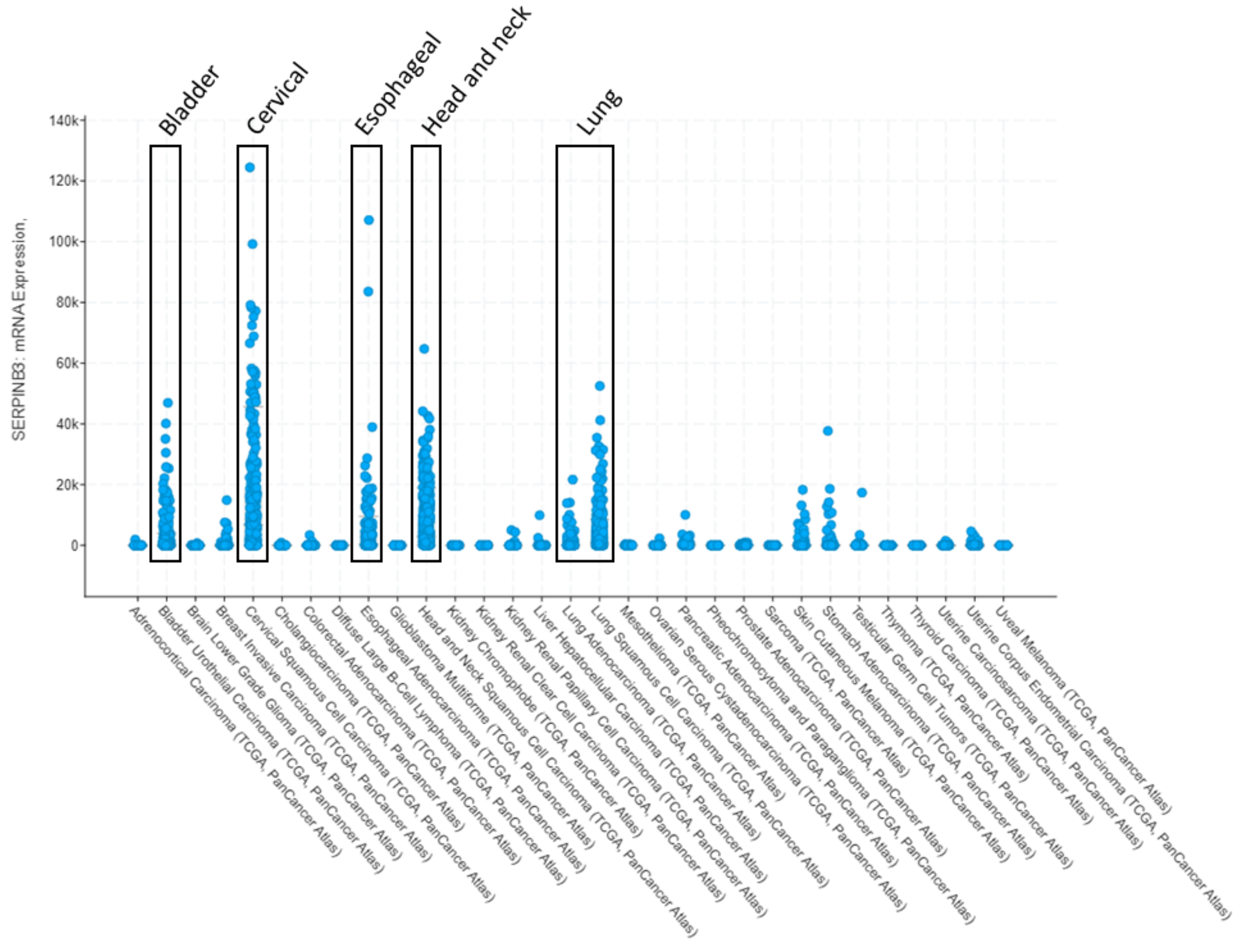

(E) Relative expression levels of SERPINB3 across all cancer types were analyzed using TCGA PanCancer Atlas RNAseq and graph was generated using cBioPortal.

F

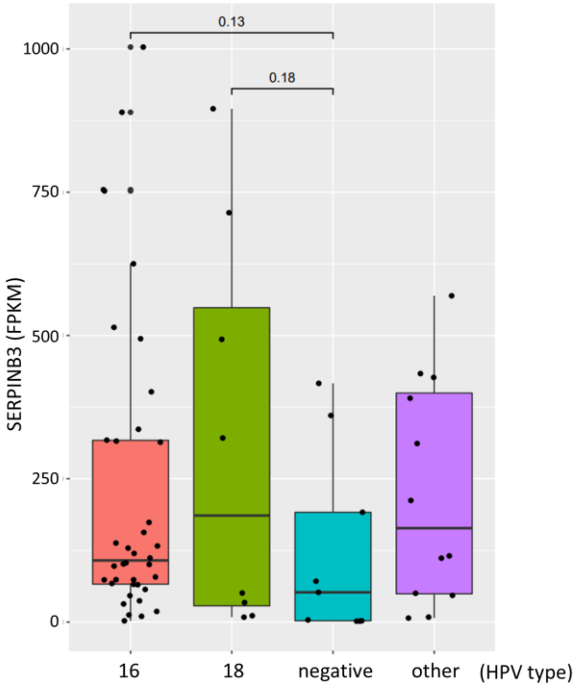

(F) SERPINB3 transcript levels in different HPV subtypes.

Supplemental Figure 2

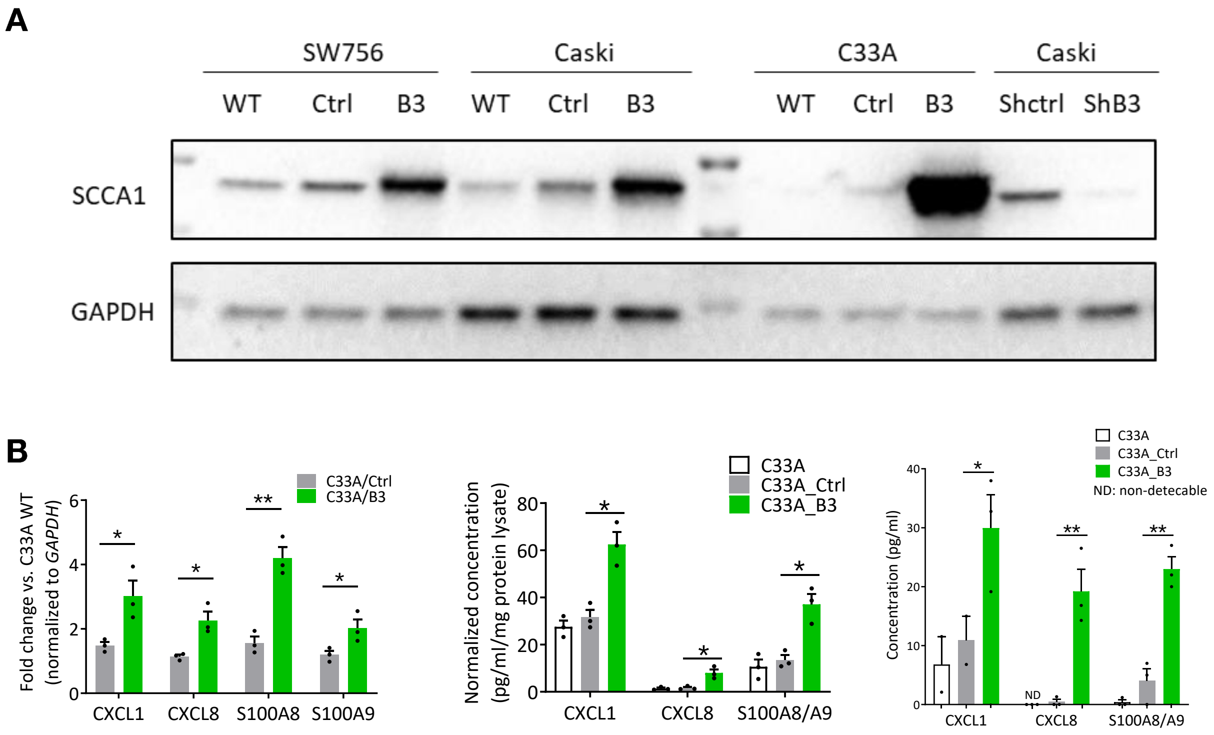

SERPINB3 expression and chemokine production. (A) Genetically modified SERPIN3 expression in cervical cancer cell lines was examined by immunoblotting. WT, wild-type parental cells; Ctrl, control vector; B3, SERPINB3-expressing vector; shctrl, scramble shRNA; shB3, shRNA targeting SERPINB3. **(B)** C33A cells were transduced with pUltra vector (C33A/Ctrl) or pUltra-SERPINB3 (C33A/B3). The upregulation of CXCL1/8 and S100A8/A9 expression was examined by qPCR. Gene expression were normalized to GAPDH and fold changes were calculated by comparing to expression levels in parental cells (C33A WT). Chemokine protein expression in cell lysate and secretion was detected by ELISA. Data are presented as mean ± SEM of n = 3 independent experiments, *$P < 0.05$, **$P < 0.01$ using Mann-Whitney test (B, left) or one-way ANOVA with Tukey's post hoc test (B, middle and right).

Supplemental Figure 3

A

B

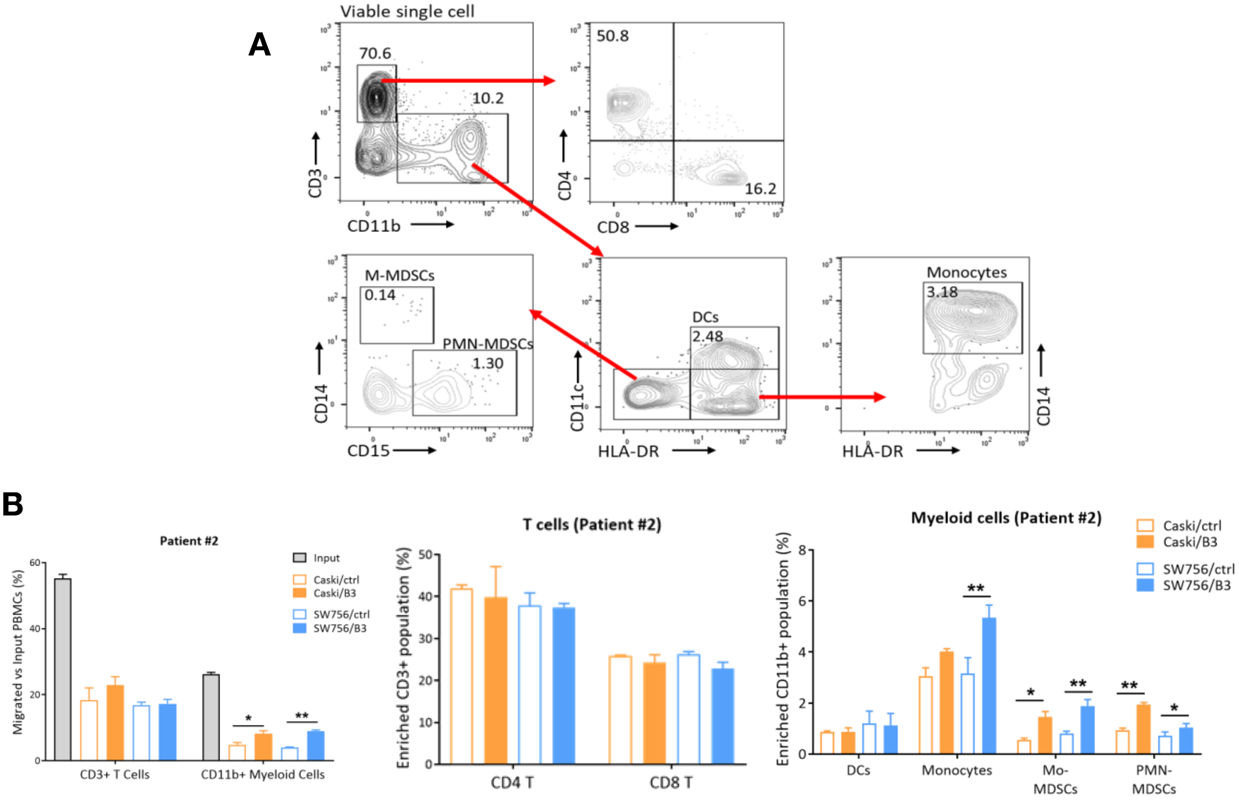

C

Migrated cells	Fold change ± SD (B3 vs Ctrl) n=7	
	Caski/B3 vs Caski/Ctrl	SW756/B3 vs SW756/B3
**CD3 T cells**	1.12 ± 0.24	0.96 ± 0.12
CD4 T	1.04 ± 0.15	0.92 ± 0.10
CD8 T	0.97 ± 0.08	0.97 ± 0.06
**CD11b Myeloid cells**	1.85 ± 0.32	2.05 ± 0.37
DCs	1.25 ± 0.21	1.5 ± 0.47
Monocytes	1.77 ± 0.36	1.8 ± 0.36
Mo-MDSCs	2.29 ± 0.39	2.0 ± 0.34
PMN-MDSCs	1.88 ± 0.55	1.69 ± 0.43

Migrated PBMC population in transwell assays. (A) Migrated immune cell populations were identified according to the reported gating strategy, including CD3+CD4+ T lymphocytes, CD3+CD8+ T lymphocyts, CD11b+HLADR+CD11c+ dendritic cells (DCs), CD11b+HLADR+CD14+ monocytes, CD11b+HLADR-CD14+ monocytic myeloid-derived suppressor cells (M-MDSCs), and CD11b+HLADR-CD15+ polymorphonucler myeloid-derived suppressor cells (PMN-MDSCs). **(B)** Representative transwell results of 7 independent experiment from 7 individual donors are shown. Each experiment was performed in duplicate transwell assays. Cell migration was calculated as a percentage of the total input PBMCs (left). Enriched migrated cell population was shown as a percentage of specifically phenotyped cells in the total migrated PBMCs (middle and right). mean ± SEM are shown, $*P < 0.05$, $**P < 0.01$ using Mann-Whitney test. **(C)** The table shows the average fold changes from 7 independent experiment of migrated cell population in Caski/B3 or SW756/B3 compared to Caski/Ctrl or SW756/Ctrl supernatant. Data are shown as mean ± SEM.

Supplemental Figure 4

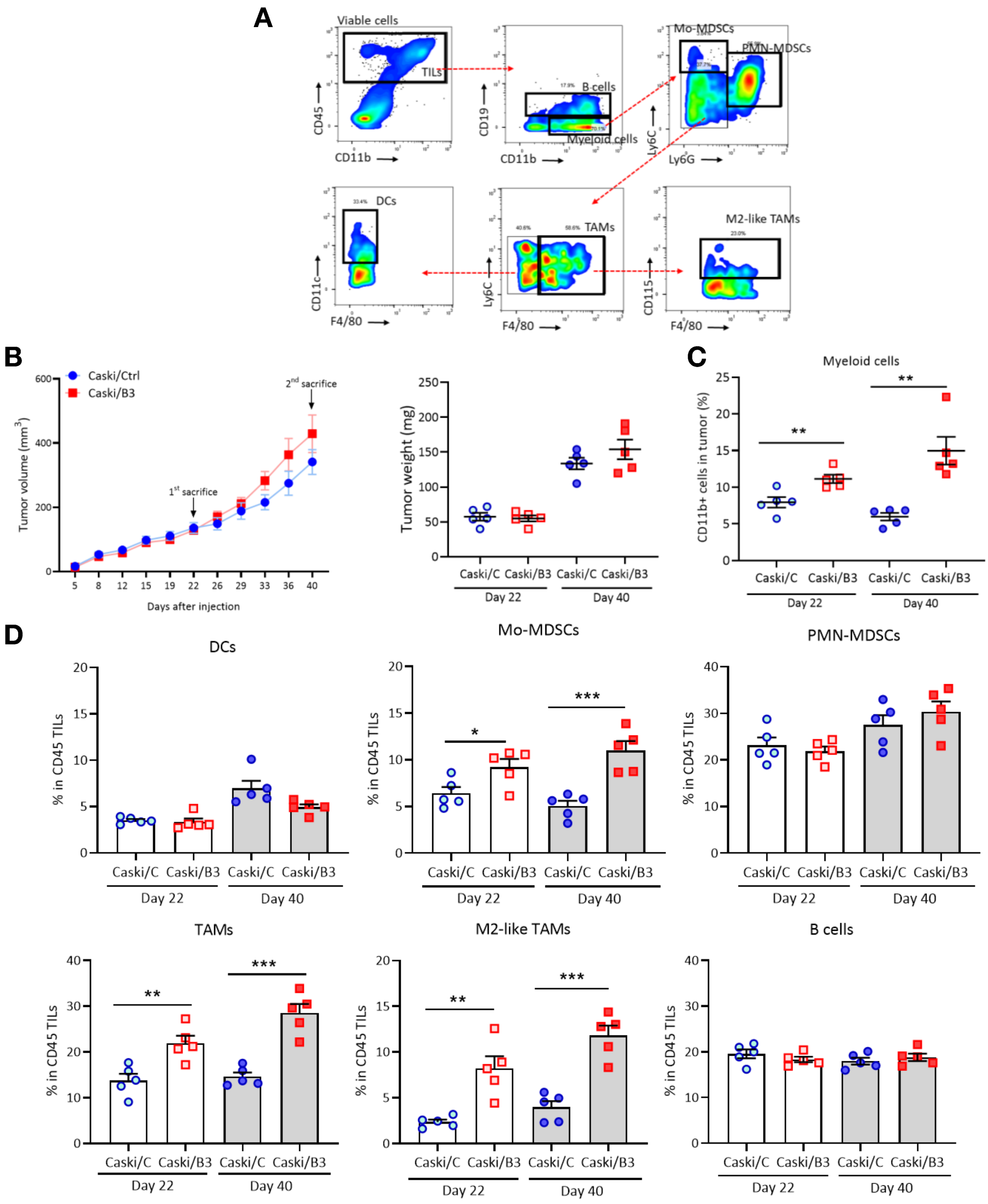

Myeloid cell infiltration in Caski xenograft tumor models. (A) Immune cell populations in Caski/Ctrl and Caski/B3 tumors were identified according to the reported gating strategy, including CD11b+Ly6G-Ly6Chigh Mo-MDSCs, CD11b+Ly6G+ PMN-MDSCs, CD11b+Ly6G-F4/80+ TAMs, CD11b+Ly6G-F4/80+CD115+ M2 macrophages, and CD11b+Ly6G-F4/80-CD11c+ DCs. **(B)** Left: Tumor growth curves of nude mice with Caski/Ctrl tumors (blue line) and Caski/B3 tumors (red lines). Right: Tumor weight at the experimental endpoint. Data are shown as mean ± SEM with individual data points, n=5 in each group. **(C)** Percentages of CD11b+ myeloid cells in tumors were analyzed by flow cytometry. **(D)** Percentages of each myeloid cell subset in total CD45+ tumor infiltrating leukocytes (TILs) were determined by flow cytometry, according to the gating strategy shown in figure S4A. The graphs represent mean ± SEM, with individual data points; *$P < 0.05$, **$P < 0.01$, ***$P < 0.00101$ using Mann-Whitney test.

Supplemental Figure 5

A

Genes	mGapdh		mCxcl1		mCxcl3		mCxcl10	
Cells	Ct1	Ct2	Ct1	Ct2	Ct1	Ct2	Ct1	Ct2
TC1 #1	17.6	17.0	38.9	39.1	37.1	38.1	29.1	28.3
TC1 #2	17.8	17.9	38.8	38.5	35.7	36.2	27.7	27.7
LL2 #1	17.6	17.6	26.6	26.4	29.1	28.6	21.9	21.0
LL2 #2	17.5	17.7	26.1	26.0	29.5	29.3	23.0	23.2

B

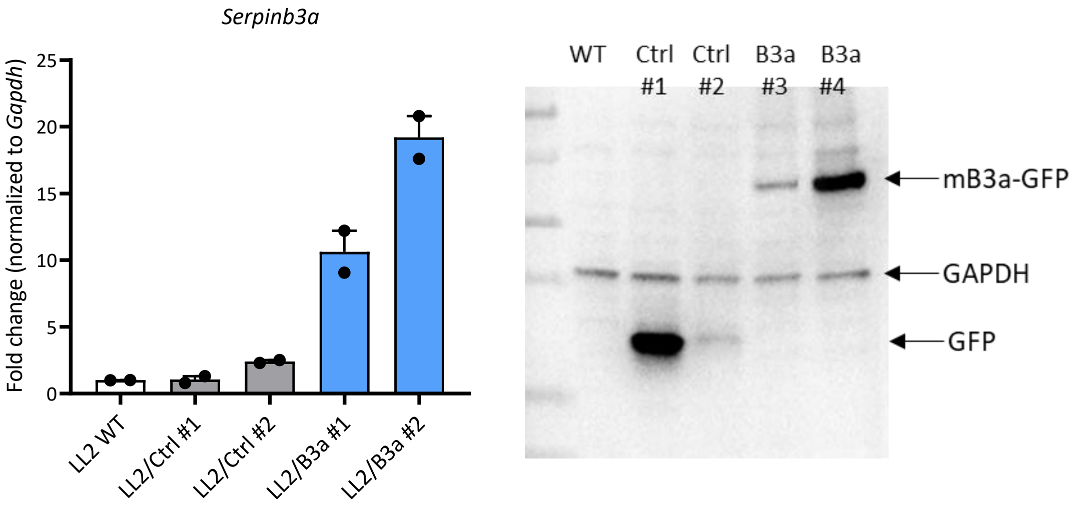

C

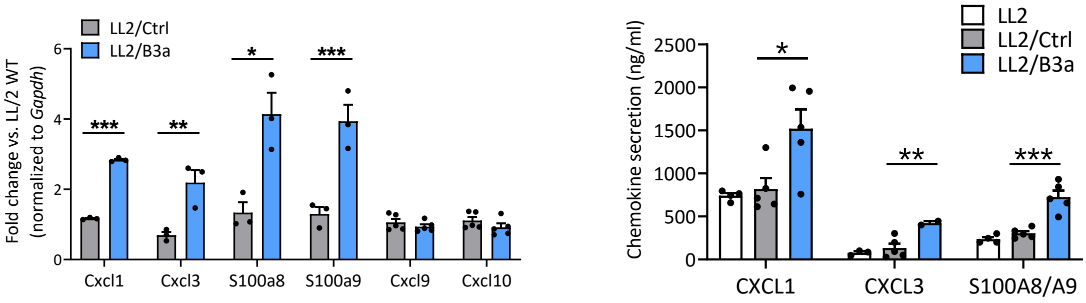

Murine chemokine and Serpinb3a expression. (A) Chemokine mRNA expression in TC1 and LL2 cells was examined by qPCR. Expression levels were shown as Ct values for each reference gene in all samples. **(B)** Murine Serpinb3a expression in LL2 cells transduced with pLV-C-GFPSpark vector with mSerpinb3a sequence (LL2/B3a) or a control vector (LL2/Ctrl) were confirmed by qPCR (left). Gene expression was normalized to mGapdh and fold changes were calculated by comparing to the expression levels in LL2 parental cells. Murine Serpinb3a-GFP-fusion protein expression was examined by immunoblotting using anti-GFP antibody (right). Ctrl#1 and B3a#4 shown comparable levels of fusion GFP expression were selected for *in vivo* tumor models. **(C)** Chemokine expression in LL2 cells transduced with pLV-C-GFPSpark vector with mSerpinb3a expression (LL2/B3a) or a control pLV-C-GFPSpark vector (LL2/Ctrl) were examined by qPCR (left) and ELISA (right). Gene expression was normalized to mGapdh and fold changes were calculated by comparing to the expression levels in LL2 parental cells. Data are shown as mean ± SEM of n = 4 independent experiments, $*P < 0.05$, $**P < 0.01$, $***P < 0.001$ using Mann-Whitney test.

Supplemental Figure 6

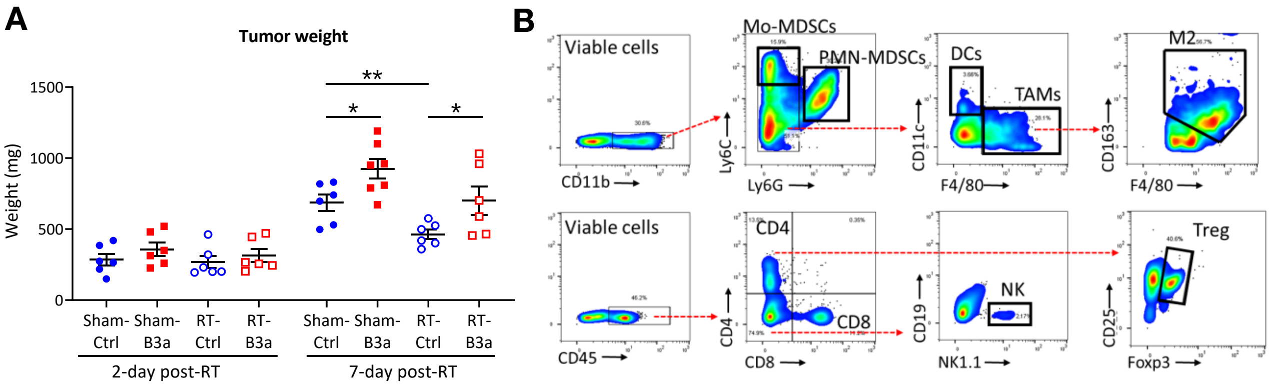

Immune cells in LL2 tumors. (A) Tumor weights at 2-day and 7-day post-RT. Data are shown as mean ± SEM and each dot represents a biologically independent animal; *$P < 0.05$, **$P < 0.01$ using one-way ANOVA with Tukey's post hoc test. **(B)** Immune cell populations in LL2/Ctrl and LL2/B3 tumors were identified according to the reported gating strategy, including CD11b+Ly6G-Ly6Chigh Mo-MDSCs, CD11b+Ly6G+ PMN-MDSCs, CD11b+Ly6G-F4/80+ TAMs, CD11b+Ly6G-F4/80+CD163+ M2 macrophages, CD45+CD4+ T cells, CD45+CD8+ T cells, and CD45+CD4+CD25+FoxP3+ Treg cells.

Supplemental Figure 7

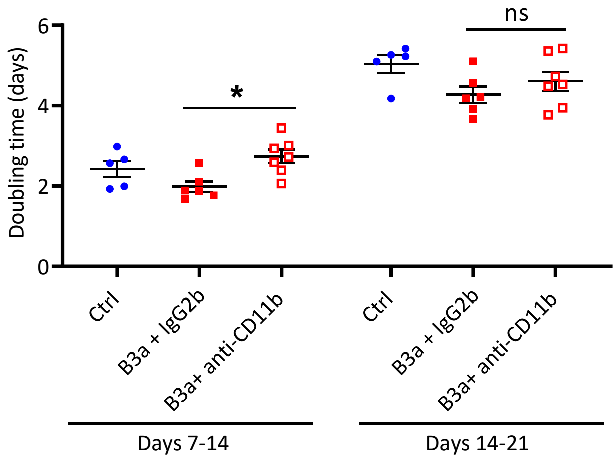

Tumor doubling time. Doubling time (DT) from days 7-14 and days 14-21 was calculated by dividing the natural logarithm of 2 by the exponent of growth. DT=durationxln2/ln(v2/v1). Data are shown as mean ± SEM and each dot represents a biologically independent animal; ns, not significant; *$P < 0.05$ using one-way ANOVA with Tukey's post hoc test.

Supplemental Figure 8

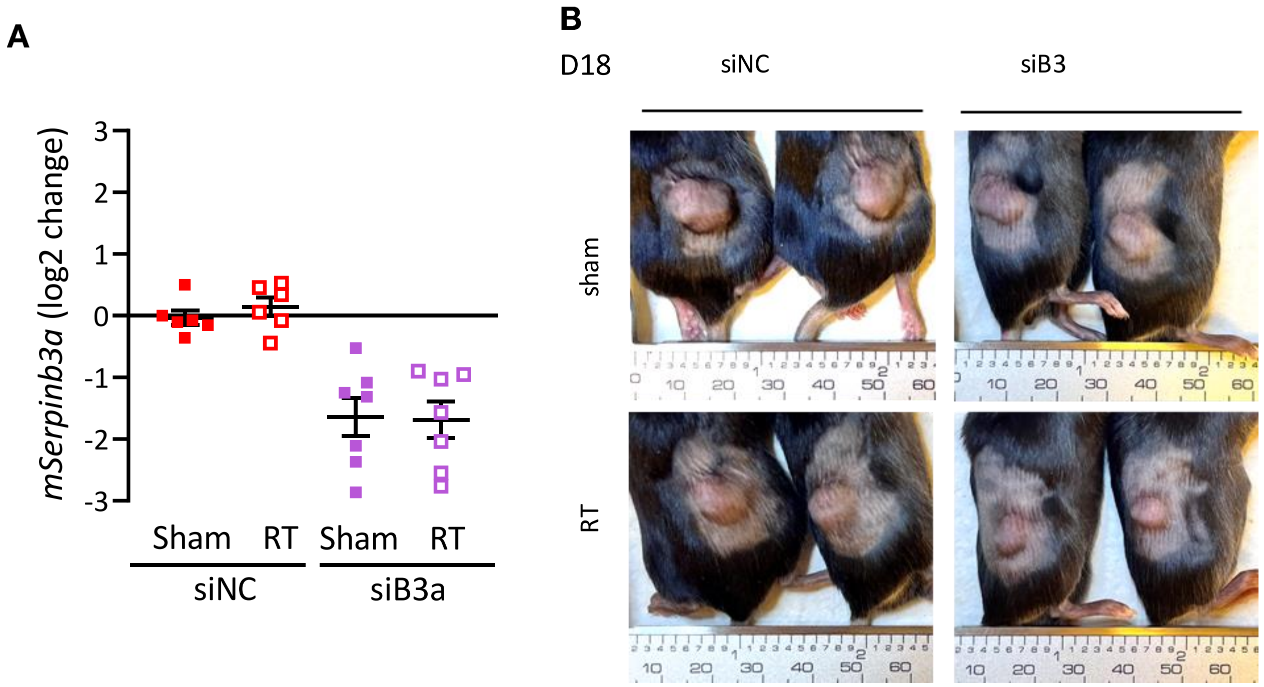

Targeting SERPINB3 in tumors. (**A**) Knockdown of *Serpinb3a* in tumors was examined by qPCR. Gene expression were normalized to *mGapdh* and shown as log2 fold change. (**B**) Images of tumor sizes on day 18. siNC: LL2/B3 treated with negative control siRNA; siB3: LL2/B3 treated with Serpinb3a siRNA; sham: without radiation treatment, RT: radiation.

Supplemental Figure 9

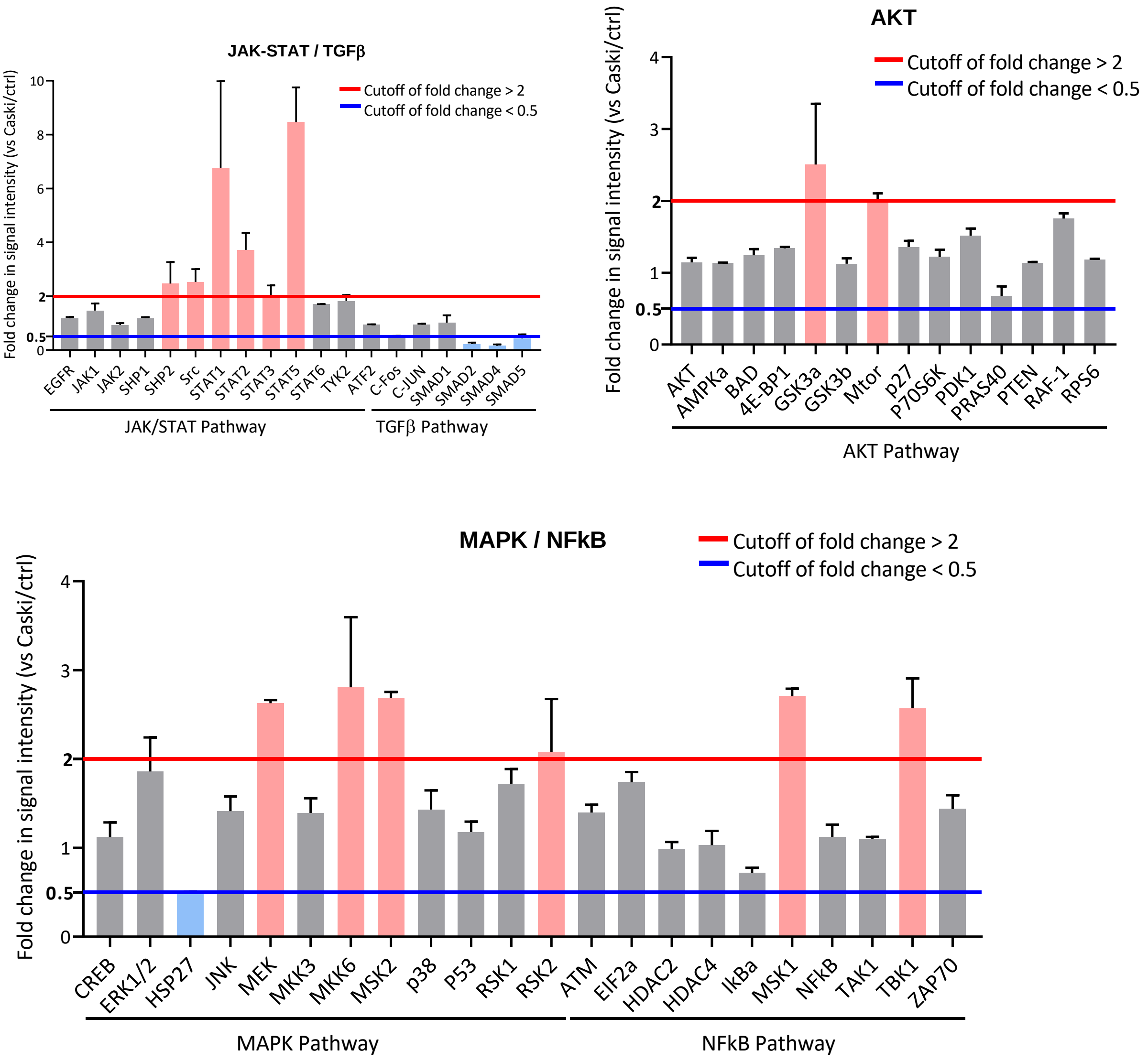

Phosphorylation protein array. Human phosphorylation pathway profiling array was used to examine the activation of MAPK, AKT, JAK/STAT, NFκB, and TGFβ signaling. 1 mg of proteins from cell lysate was used for incubation with phosphorylation antibody array. Fold changes in phosphorylation were calculated by normalizing the intensity of both Caski/Ctrl and Caski/B3 to the basal levels in Caski parental cells and comparing the phosphorylation intensity in Caski/B3 to the levels in Caski/Ctrl cells. Red line indicates fold change ≥ 2 and blue line indicates fold change ≤ 0.5.

Supplemental Figure 10

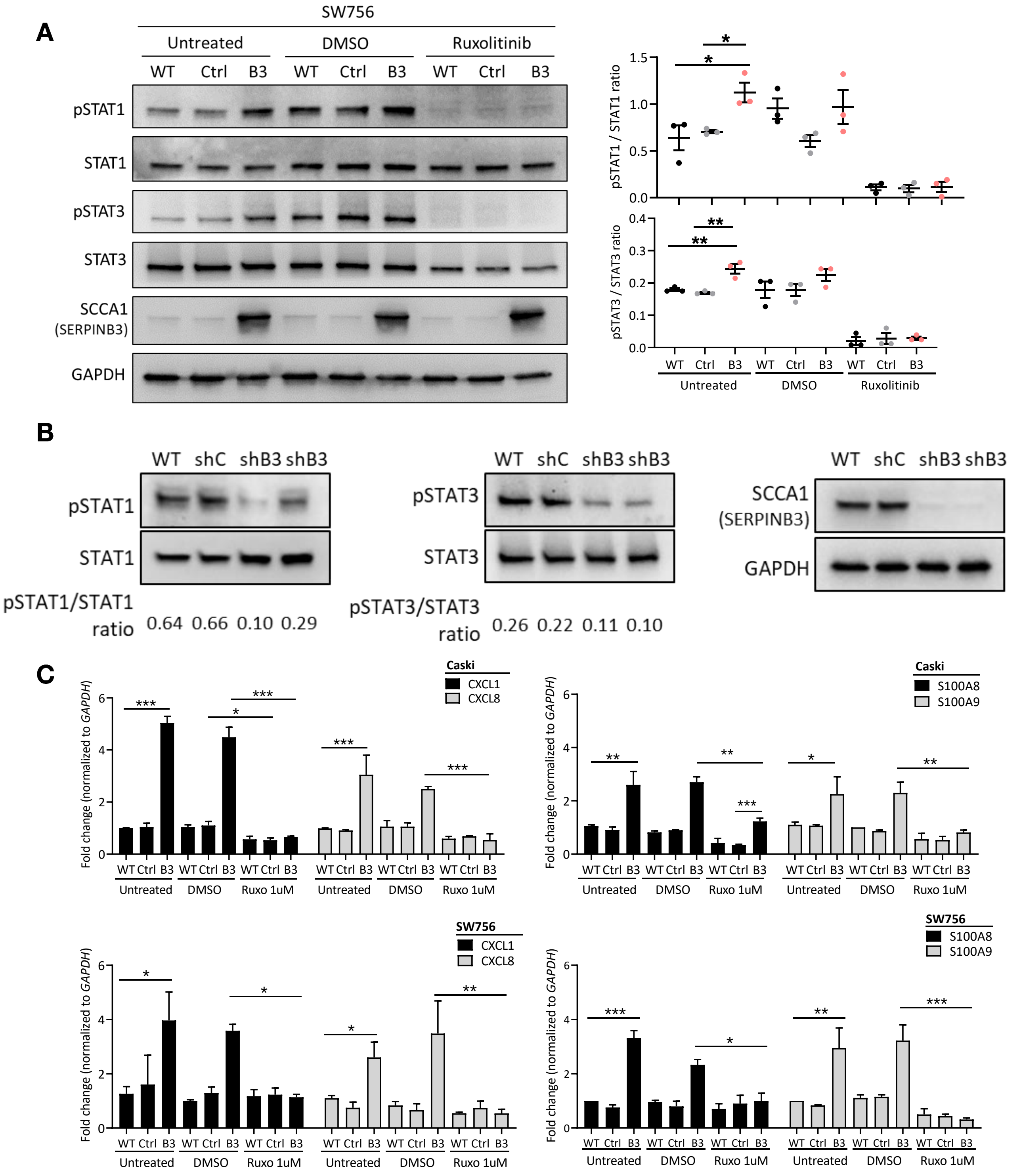

STAT activation in SERPINB3 cells. (A) Immunoblotting (left) and quantification (right) show the inhibition of STAT1/3 phosphorylation after treating SW756 parental cells (WT), SW756/Ctrl (C), and SW756/B3 (B3) with 1uM Ruxolitinib for 48 h. **(B)** Caski cells were transfected with scramble shRNA (shC) or SERPINB3 shRNA (shB3). Immunoblotting shows the reduced STAT1/3 phosphorylation by the knockdown of SERPINB3. **(C)** Caski/SW756 cells were treated with 1uM Ruxolitinib and the expression CXCL1/8 and S100A8/A9 mRNA was examined by qPCR. Gene expression were normalized to GAPDH and fold changes were calculated by comparing to the expression levels in parental cells (WT). Data are shown as mean ± SEM of n = 3, *$P < 0.05$, **$P < 0.01$, ***$P < 0.001$ using one-way ANOVA with Tukey's post hoc test.

Supplemental Figure 9

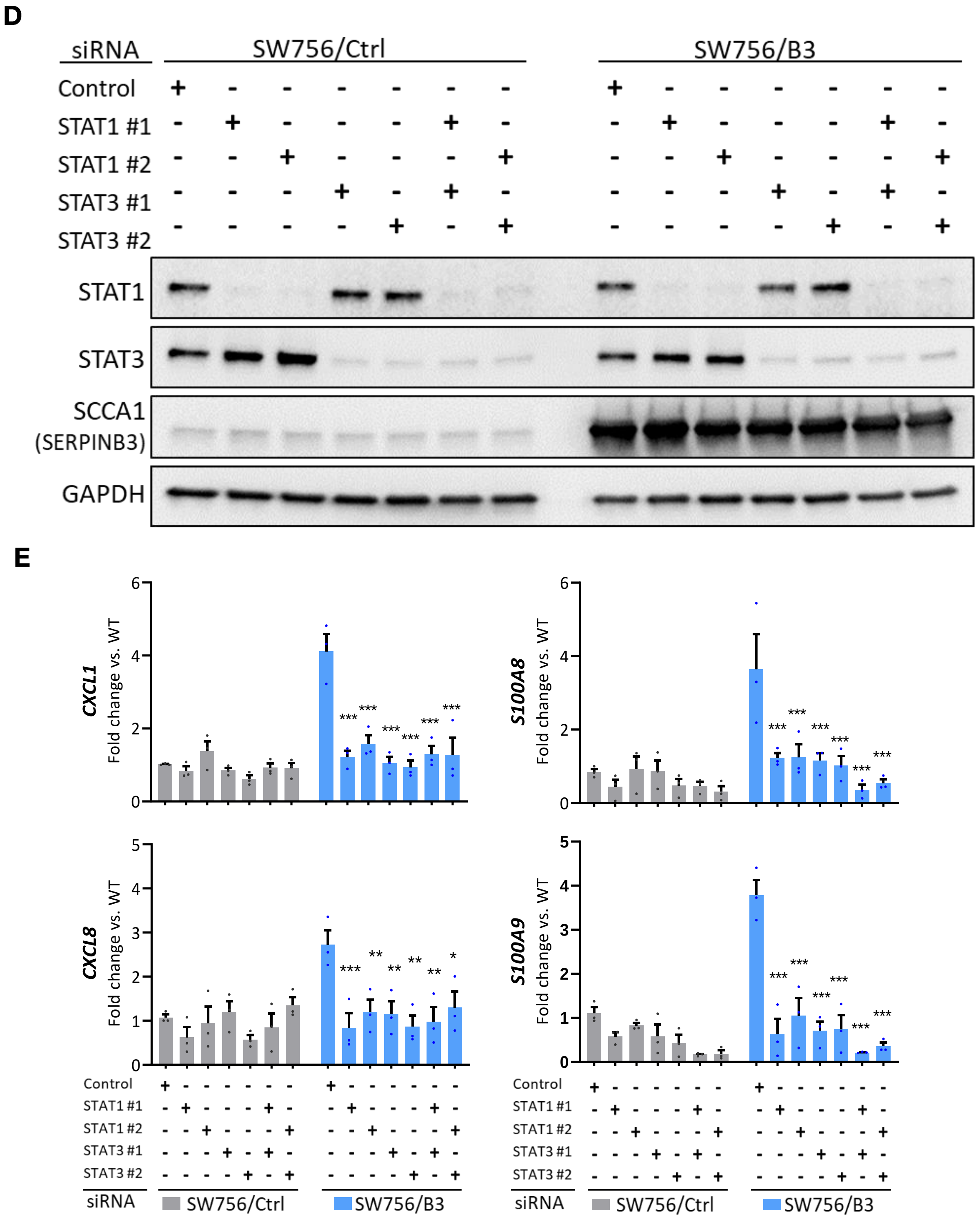

STAT activation in SERPINB3 cells. (D) Immunoblotting shows the knockdown of STAT1/3 by siRNA in SW756 cells **(E)** The expression CXCL1/8 and S100A8/A9 mRNA was examined by qPCR after STAT1/3 knockdown. Gene expression were normalized to GAPDH and fold changes were calculated by comparing to the expression levels in SW756/Ctrl transfected with negative control siRNA. Data are shown as mean ± SEM of n = 3, *P < 0.05, **P < 0.01, ***P < 0.001 using one-way ANOVA with Tukey's post hoc test.

Supplemental Figure 11

A

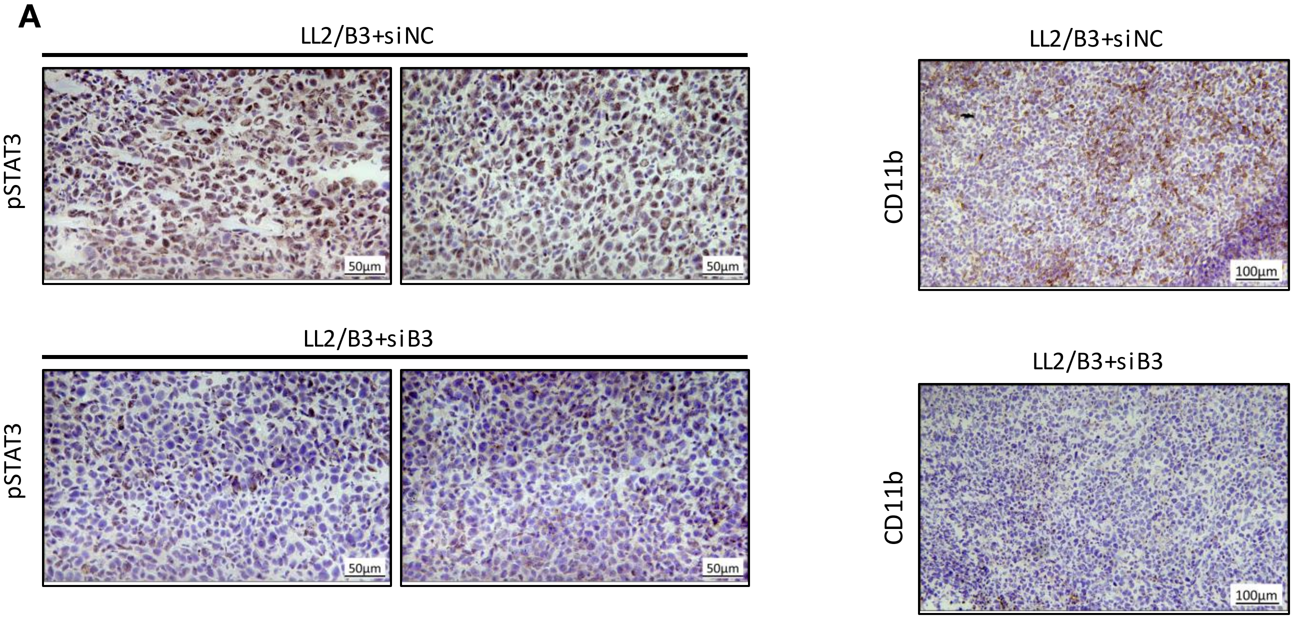

(A) Representative immunohistochemistry images of mouse tumors treated with negative control (siNC) or Serpinb3a siRNA (siB3) stained with pSTAT3 and CD11b.

B

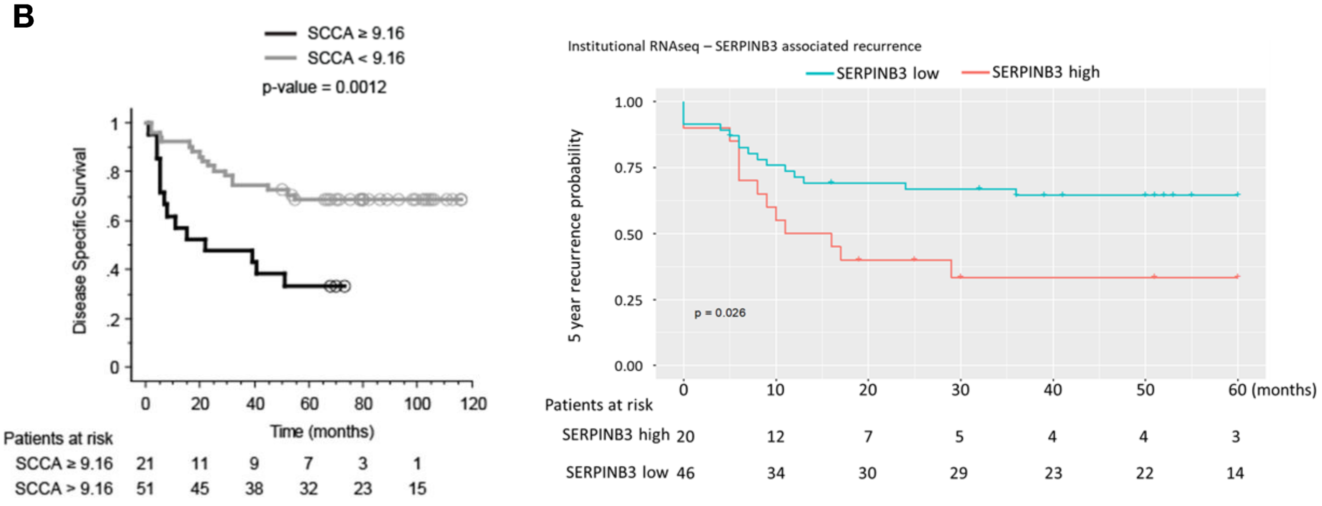

(B) SCCA prognostic value. Left: Kaplan-Meier plot show overall survival in patients with serum SCCA <9.16 ng/ml, compared to patients with serum SCCA ≥9.16 ng/ml. The average pretreatment serum SCCA value of 9.16 ng/ml from 72 cancer patients was used as a cutoff. **Right:** Recurrence probability of patients with high and low SERPINB3 transcripts from RNAseq cohort.

Supplemental Table 1. Patient characteristics

	SERPINB3/Low		SERPINB3/Intermediate		SERPINB3/H		TMA cohort
	R	NR	R	NR	R	NR	
**Patients**	11	11	6	16	12	10	72
**Age (months)**							
Median	49	53	66.5	57	51.5	53.5	52
Range	43-74	34-77	50-78	25-81	42-72	32-67	27-85
**Race**							
Asian	1 (9.1%)	0	0	2 (12.5%)	0	0	3 (4.2%)
Black	3 (27.3%)	3 (27.3%)	3 (50%)	2 (12.5%)	1 (8.3%)	2 (20%)	14 (19.4%)
White	7 (63.6%)	7 (63.6%)	3 (50%)	11 (68.8%)	11 (91.7%)	8 (80%)	54 (75%)
Hispanic	0	1 (9.1%)	0	1 (6.3%)	0	0	1 (1.4%)
**FIGO Stage (2018)**							
I	1 (9.1%)	5 (45.5%)	0	2 (12.5%)	2 (16.7%)	1 (10%)	11 (15.3%)
II	2 (18.2%)	3 (27.3%)	2 (33.3%)	3 (18.8%)	1 (8.3%)	1 (10%)	19 (26.4%)
III	5 (45.5%)	3 (27.3%)	2 (33.3%)	10 (62.5%)	8 (66.7%)	8 (80%)	37 (51.4%)
IV	3 (27.3%)	0	2 (33.3%)	1 (6.3%)	1 (8.3%)	0	5 (6.9%)
**Lymph Node Involvement**							
supraclavicular	1 (9.1%)	0	1 (16.7%)	0	1 (8.3%)	0	1 (1.4%)
Aortic	2 (18.2%)	0	1 (16.7%)	0	3 (25%)	1 (10%)	7 (9.7%)
Pelvic	3 (27.3%)	1 (9.1%)	1 (16.7%)	6 (37.5%)	4 (33.3%)	7 (70%)	24 (33.3%)
None	5 (45.5%)	10 (90.9%)	2 (33.3%)	10 (62.5%)	4 (33.3%)	2 (20%)	40 (55.6%)
**Follow up times (month)**							
Median	44	86	28	105.5	16.5	79.5	67
Range	2-117	62-119	2-115	5-119	1-102	19-113	1-119
**Histology**							
Squamous	7 (63.6%)	6 (54.5%)	5 (83.3%)	14 (87.5%)	10 (83.3%)	10 (100%)	61 (84.7%)
Adenocarcinoma	2 (18.2%)	5 (45.5%)	0	1 (6.3%)	1 (8.3%)	0	6 (8.3%)
Adenosquamous	0	0	1 (16.7%)	1 (6.3%)	0	0	1 (1.4%)
Other*	1 (9.1%)	0	0	0	1 (8.3%)	0	4 (5.6%)
**Chemotherapy**	11 (100%)	11 (100%)	5 (83.3%)	16 (100%)	11 (91.7%)	9 (90%)	66 (91.7%)
**Treatment Intent**							
Curative	9 (81.8%)	11 (100%)	3 (50%)	16 (100%)	11 (91.7%)	10 (100%)	67 (93.1%)
Palliative	2 (18.2%)	0	2 (33.3%)	0	1 (8.3%)	0	1 (1.4%)
Post-op	0	0	0	0	0	0	2 (2.8%)
Surgery Only	0	0	0	0	0	0	1 (1.4%)
None	0	0	1 (16.7%)	0	0	0	1 (1.4%)
**Serum SCCA (ng/ml)**							
Median	1.3	1.45	1.95	2.9	9.65	9.7	2.9
Range	0-17.1	0-4.3	0-60	0-26.3	0-220	1.6-25.5	0-66.6
**HPV Types**							
16	3	7	4	11	5	7	N/A
18	3	1	0	0	2	1	N/A
negative	3	2	0	2	2	0	N/A
Other†	2	1	2	3	3	2	N/A
**Recurrence**	11	-	6	-	12	-	35
**Non-recurrence**	-	11	-	16	-	10	37

R, recurrence; NR, non-recurrence; N/A, not available

*Others: leiomyosarcoma, malignant mixed mullerian tumors, small cell

†Other: HPV Types 33, 45, 52, 56, 58, 59, 66

Supplemental Table 2.

Recurrence free survival

Variable	Univariate Cox regression		Multivariate Cox regression	
	HR (95%CI)	p-value	covariate-adjusted HR (95% CI)	p-value
Age (continuous)	0.979 (0.9519 - 1.007)	0.137	0.9727 (0.9439 - 1.002)	0.0709
FIGO		0.9442		0.01[#]
I-II (reference)	-	-	-	-
III	1.176 (0.5022 - 2.753)	0.7092	1.0822 (0.4595 - 2.548)	0.8566
IV	5.51 (1.5949 - 19.039)	0.0069	8.9726 (2.3639 - 34.057)	0.0013
Race			-	-
White	-	-		-
non-White	0.8125 (0.3774 - 1.749)		0.9255 (0.3830 - 2.237)	0.8635
SCCA/pSTAT3		0.0487*		0.0199[#]
Low/Low(reference)	-	-	-	-
Low/High or High/High	1.874 (0.8645 - 4.061)	0.112	2.2822 (0.9781 - 5.325)	0.0563
High/Low	1.367 (1.3267 - 11.593)	0.0135	5.5454 (1.6563 - 18.566)	0.0055

Cancer specific survival

Variable	Univariate Cox regression		Multivariate Cox regression	
	HR (95%CI)	p-value	covariate-adjusted HR (95% CI)	p-value
Age (continuous)	0.9742 (0.9623 - 1.007)	0.124	0.9624 (0.9276 - 0.9985)	0.0413
FIGO				
I-II (reference)	-		-	-
III	1.121 (0.4105 - 3.061)	0.824	0.9637 (0.3489 - 47.9586)	0.943
IV	7.124 (1.8927 - 26.815)	0.0037	14.863 (3.49 - 63.35)	0.0003
Race				
White				
non-White	0.5317 (0.1993 - 1.419)	0.207	0.71 (0.22 - 2.28)	0.563
SCCA/pSTAT3				
Low/Low(reference)	-		-	
Low/High or High/High	2.398 (0.921 - 6.243)	0.0732	2.8384 (0.96 - 8.3679)	0.059
High/Low	7.335 (2.209 - 24.359)	0.0011	11.6529 (2.8314 - 47.9586)	0.0007

Likelihood ratio test P value

Supplemental Table 3.

**Antibodies**
Human Antibodies (all from BioLegend unless noted otherwise)
Pacific Blue anti-human CD3 [SK7, 344824]<br>PE/Cyanine7 anti-human CD4 [RPA-T4, 300512]<br>PerCP anti-human CD8 [SK1, 344708]<br>PE anti-human CD11b [ICRF44, 301305]<br>APC anti-human CD11c [3.9, 301613]<br>FITC anti-human HLA-DR [L243, 307603]<br>CD14-PerCP-Vio 700, human [Tuk4, 130-113-713, MACS]<br>PE/Cy7 anti-human CD15 (SSEA-1) [W6D3, 323029]
Mouse Antibodies (all from BioLegend unless noted otherwise)
Pacific Blue anti-mouse CD45 [30-F11, 103126]<br>CD45-PE, mouse [REA737, 130-110-797, MACS]<br>Brilliant Violet 510 anti-mouse CD3 [17A2, 100233]<br>PE anti-mouse CD4 [GK1.5, 100407]<br>PE/Cy7 anti-mouse CD8a [53-6.7, 100721]<br>FITC anti-mouse CD25 [PC61, 102005]<br>PE anti-mouse F4/80 [BM8, 123110]<br>APC anti-mouse CD163 [S15049I, 115305]<br>FITC anti-mouse Ly-6C [HK1.4, 128005]<br>PF/Cyanine7 anti-mouse Ly-6G [1A8, 127617]<br>Ki-67 antibody, anti-human/mouse, FITC, REAfinity [REA183, 130-117-803, MACS]<br>TNF-a antibody, anti-mouse, FITC, REAfinity [REA636, 130-124-212, MACS]<br>IFN-g antibody, anti-mouse, APC, REAfinity [REA638, 130-123-283, MACS]<br>CD11b antibody, anti-mouse, VioBlue, REAfinity [REA592, 130-113-810, MACS]<br>Brilliant Violet 510 anti-mouse/human CD11b [M1/70, 101245]<br>PerCP/Cyanine5.5 anti-mouse CD115 CSF-1R [AFS98, 135525]<br>FoxP3 Antibody, anti-mouse, APC, REAfinity [REA788, 130-111-679, MACS]<br>FITC anti-human/mouse Granzyme B [GB11, 515403]<br>Pacific Blue anti-mouse Perforin [S16009B, 154407]
Antibodies for immunoblotting
Phospho-Stat1 (Tyr701) (58D6) Rabbit mAb [9167, Cell Signaling]<br>Stat1 p84/p91 (C-136) HRP, mouse [sc-464, Santa Cruz Biotechnology]<br>p-STAT3 HRP, mouse antibody (B-7) [sc-8059, Santa Cruz Biotechnology]<br>Stat3 (124J6) mouse [9139S, Cell Signaling]<br>HRP-conjugated GAPDH mouse [HRP-6004, Proteintech]<br>Serpin B3/SCCA1 antibody (2F5), mouse [H00006317-M01, Novus Biologicals]<br>GFP (B-2), mouse [sc-9996, Santa Cruz Biotechnology]<br>Jak1 (D1T6W) mouse mAb [50996, Cell Signaling]<br>Lamin A/C (4C11) Mouse mAb [4777, Cell Signaling]

Supplemental Table 4.

Primers	Forward	Backward
human S100A8	AGACCGAGTGTCCTCAGTATATC	TGCCACGCCCATCTTTATC
human S100A9	GCTGGAACGCAACATAGAGA	TCGCACCAGCTCTTTGAAT
human CXCL1	GGATTGTGCCTAATGTGTTTGAG	GACAGTGTGCAGGTAGAGTTAAT
human CXCL8	CTTGGCAGCCTTCCTGATTT	GGGTGGAAAGGTTTGGAGTATG
human GAPDH	CTGGGCTACACTGAGCACC	AAGTGGTCGTTGAGGGCAATG
human SERPINB3	CGCGGTCTCGTGCTATCTG	ATCCGAATCCTACTACAGCGG
mouse CXCL9	CAGGCTAGGAGTGGTGAAATG	CAGAGGCCAGAAGAGAGAAATG
mouse CXCL10	TCAGGCTCGTCAGTTCTAAGT	CCTTGGGAAGATGGTGGTTAAG
mouse CXCL1	CGAAGTCATAGCCACACTCAA	GAGCAGTCTGTCTTCTTTCTCC
mouse SERPINB3A	CATCAGCACAGATAGCAGAAGA	AGGAGATTCTGCCAAAGAAGAG
mouse CXCL3	GATACTGAAGAGCGGCAAGT	CAGGTAAAGACACATCCAGACA
mouse S100A8	CTTTGTCAGCTCCGTCTTCA	TGTAGAGGGCATGGTGATTTC
mouse S100A9	CTGGGCTTACACTGCTCTTAC	GGTGTCGATGATGGTGGTTAT
mouse GAPDH	AACAGCAACTCCCACTCTTC	CCTGTTGCTGTAGCCGTATT
